# Bafilomycin A1 triggers proliferative potential of senescent cancer cells *in vitro* and in NOD/SCID mice

**DOI:** 10.18632/oncotarget.14066

**Published:** 2016-12-21

**Authors:** Halina Was, Kamila Barszcz, Joanna Czarnecka, Agata Kowalczyk, Tytus Bernas, Ewelina Uzarowska, Paulina Koza, Agata Klejman, Katarzyna Piwocka, Bozena Kaminska, Eva Sikora

**Affiliations:** ^1^ Laboratory of Molecular Basis of Ageing, Nencki Institute of Experimental Biology, Polish Academy of Sciences, 02-093 Warsaw, Poland; ^2^ Laboratory of Molecular Neurobiology, Nencki Institute of Experimental Biology, Polish Academy of Sciences, 02-093 Warsaw, Poland; ^3^ Laboratory of Cytometry, Nencki Institute of Experimental Biology, Polish Academy of Sciences, 02-093 Warsaw, Poland; ^4^ Laboratory of Imaging Tissue Structure and Function, Nencki Institute of Experimental Biology, Polish Academy of Sciences, 02-093 Warsaw, Poland; ^5^ Laboratory of Animal Models, Nencki Institute of Experimental Biology, Polish Academy of Sciences, 02-093 Warsaw, Poland; ^6^ Laboratory of Neurobiology, Nencki Institute of Experimental Biology, Polish Academy of Sciences, 02-093 Warsaw, Poland

**Keywords:** colon cancer, chemotherapy, senescence, autophagy, angiogenesis

## Abstract

Anticancer therapies that induce DNA damage tend to trigger senescence in cancer cells, a process known as therapy-induced senescence (TIS). Such cells may undergo atypical divisions, thus contributing to tumor re-growth. Accumulation of senescent cancer cells reduces survival of patients after chemotherapy. As senescence interplays with autophagy, a dynamic recycling process, we sought to study whether inhibition of autophagy interferes with divisions of TIS cells. We exposed human colon cancer HCT116 cells to repeated cycles of a chemotherapeutic agent – doxorubicin (doxo) and demonstrated induction of hallmarks of TIS (e.g. growth arrest, hypertrophy, poliploidization and secretory phenotype) and certain properties of cancer stem cells (increased NANOG expression, percentages of CD24+ cells and side population). Colonies of small and highly proliferative progeny appeared shortly after drug removal. Treatment with bafilomycin A1 (BAF A1), an autophagy inhibitor, postponed short term *in vitro* cell re-population. It was associated with reduction in the number of diploid and increase in the number of poliploid cells. In a long term, a pulse of BAF A1 resulted in reactivation of autophagy in a subpopulation of HCT116 cells and increased proliferation. Accordingly, the senescent HCT116 cells treated with BAF A1 when injected into NOD/SCID mice formed tumors, in contrast to the controls.

Our results suggest that senescent cancer cells that appear during therapy, can be considered as dormant cells that contribute to cancer re-growth, when chemotherapeutic treatment is stopped. These data unveil new mechanisms of TIS-related cancer maintenance and re-population, triggered by a single pulse of BAF A1 treatment.

## INTRODUCTION

Colon cancer is the third most common cause of cancer-related mortality in the US [1]. Outcomes of chemotherapeutic treatment of colon cancer in clinics are hampered by low response rates, small survival benefits and severe side effects [2]. Poor outcomes are related to heterogeneity of tumors, their clonal evolution and intrinsic drug resistance of cancer cells. One of the postulated mechanisms responsible for the latter is the therapy-induced senescence (TIS). Senescence is a state of irreversible growth arrest of normal cells, when they reach the end of their replicative lifespan [3]. Conversely, a stress-induced premature senescence (SIPS), which could be triggered by oxidative stress or DNA damaging agents, is an acute, short-term and telomere shortening-independent event [4]. Apart from an irreversible growth arrest, senescent cells exhibit several morphological and biochemical changes: increased size and granularity, augmented activity of senescence-associated β-Galactosidase (SA-β-Gal), activation of the DNA damage response (DDR) and mTOR pathway, and senescence-associated secretory phenotype (SASP) [4–7]. Recent clinical studies have demonstrated that cancer cells may undergo SIPS in response to chemotherapy [8–13]. However, a growing body of evidence supports correlation between accumulation of TIS cancer cells and reduced survival of patients subjected to anticancer treatment [5, 8–11, 14]. This could be attributed to SASP-related remodeling of tumor environment [5, 15] and/or atypical senescent cell divisions [16–18]. A few studies showed that senescent cells transiently display stem cell properties [19, 20]. The cancer stem cell (CSC) model suggests that only a small subset is responsible for sustaining tumorigenesis and tumor re-growth after therapy [21]. CSCs exhibit the stem cell properties including a self-renewal and an ability to differentiate [22–24]. Quiescent or slow cycling CSCs may survive therapeutic intervention, which results in tumor relapse [21].

Non-proliferating cells are not able to redistribute and eliminate damaged organelles, proteins or aggregates through cell division [25]. Therefore, autophagy, a controlled lysosomal degradation of macromolecules and organelles [26], may be essential for senescent cells [27]. Indeed, autophagy was reported to facilitate rapid protein turnover that can trigger recycling of proteins into SASP factors [28]. Accordingly, autophagy was reported to be crucial for maintaining stemness [29]. Increasing evidence indicates the importance of autophagy in human cancer cells both for drug-induced cell death and as a self-protective mechanism [30–32]. Inhibiting autophagy to overcome resistance to chemotherapy has been investigated in five clinical phase I trials where hydroxychloroquine (HCQ) was combined with chemotherapeutics. Nonetheless, additional mechanistic studies in preclinical models are necessary to support clinical application of autophagy inhibitors [33]. In particular, relationship between TIS-associated remodeling and induction of tumorigenic capacity of senescent cancer cells needs to be investigated.

Here, we characterize properties of drug-induced senescent colon cancer cells in long term cultures. We have investigated whether inhibition of autophagy by a single pulse of bafilomycin A1 (BAF A1), a specific inhibitor of lysosomal acidification, affects their ability to re-populate *in vitro* cultures and to form tumors in NOD/SCID mice.

## RESULTS

### Senescent colon cancer HCT116 cells exhibit stem-cell like properties and re-populate culture after chemotherapeutic removal

**Figure 1:**
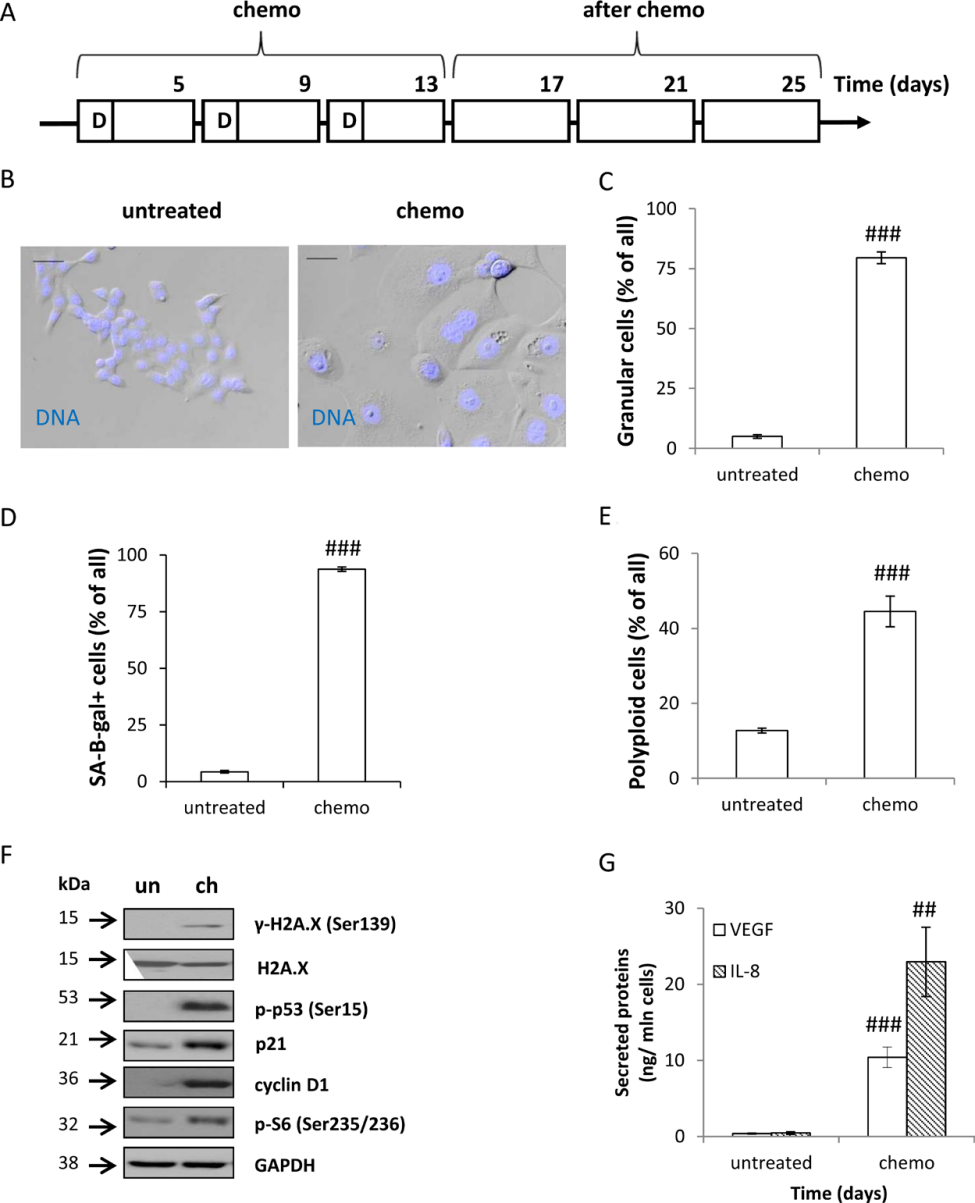
Colon cancer HCT116 cells treated with doxorubicin cycles show features of senescence (**A**) A scheme of the experiment. CHEMO protocol. Cells were subjected to three cycles of doxorubicin (D) as follows: cells were treated with 100 nM of doxorubicin for 24 hours, then the medium was removed and cells were cultured in a drug-free medium for the next 3 days. AFTER CHEMO protocol. After the 3rd doxorubicin cycle HCT116 cells were cultured in a drug-free medium for 14 days. Medium was changed every four days. Cells were examined for senescent markers on the 13th day (CHEMO). (**B**) Representative photos show morphological alterations in CHEMO-treated cells. Cell nuclei were stained with H33342 (blue). Original magnification 200×. Data were acquired with transmitted light and fluorescence microscopy. Scale bar–100 μm. (**C**) Percentages of granular cells as determined by FSC/SSC analysis using flow cytometry. (**D**) Quantification of SA-β-gal^+^ cells. Untreated or CHEMO-treated cells were cytospined and cytochemical staining for SA-β-gal activity was performed. (**E**) Percentages of polyploid cells. Cell cycle analysis was performed using PI staining and flow cytometry. (**F**) The expression of DDR and geroconversion proteins in untreated (un) or CHEMO-treated (ch) cells. Representative blots show levels of γ-H2A.X, H2A.X, p-p53, p21, cyclin D1 and p-S6 proteins. GAPDH was used as a loading control. (**G**) Secretion of VEGF and IL-8 in CHEMO-treated cultures. Cytokine levels were determined by colorimetric ELISA in supernatants harvested from untreated and treated cells. Results were normalized to total cell number counted in Bürker's chamber. Each bar represents mean ± SEM, *n* ≥ 3; ^#^*p* < 0.05, ^##^*p* < 0.01, ^###^*p* < 0.001 -untreated vs. CHEMO.

To mimic a regime of chemotherapy in patients, we subjected human colon cancer HCT116 cell cultures to long-term, repeated treatment with a chemotherapeutic drug. Cells were treated with 100 nM doxorubicin (doxo, D) for 24 hours. Following its removal, the cells were cultured in the drug-free medium for the next 3 days. The cycle was repeated three times (Figure 1A, CHEMO protocol). Subsequently, to mimic a post chemotherapy period, we cultured HCT116 cells in the drug-free medium for additional 14 days, with the medium changed every four days (Figure 1A, AFTER CHEMO protocol). On the 13th day the CHEMO-treated cells exhibited several features of senescence: flatten morphology (Figure 1B), increased size and granularity (Figure 1B, 1C, Supplementary Figure S1A), augmented SA-β-gal activity (Figure 1D, Supplementary Figure S1B) and polyploidization (Figure 1E, Supplementary Figure S1C). Moreover, the elevated expression of DDR proteins: γ-H2A.X, p-p53, and p21, and geroconversion markers [7]: cyclin D1 and p-S6 (Figure 1F) was detectable. In addition, the cells up-regulated secretion of SASP factors: VEGF and IL-8 (Figure 1G).

**Figure 2:**
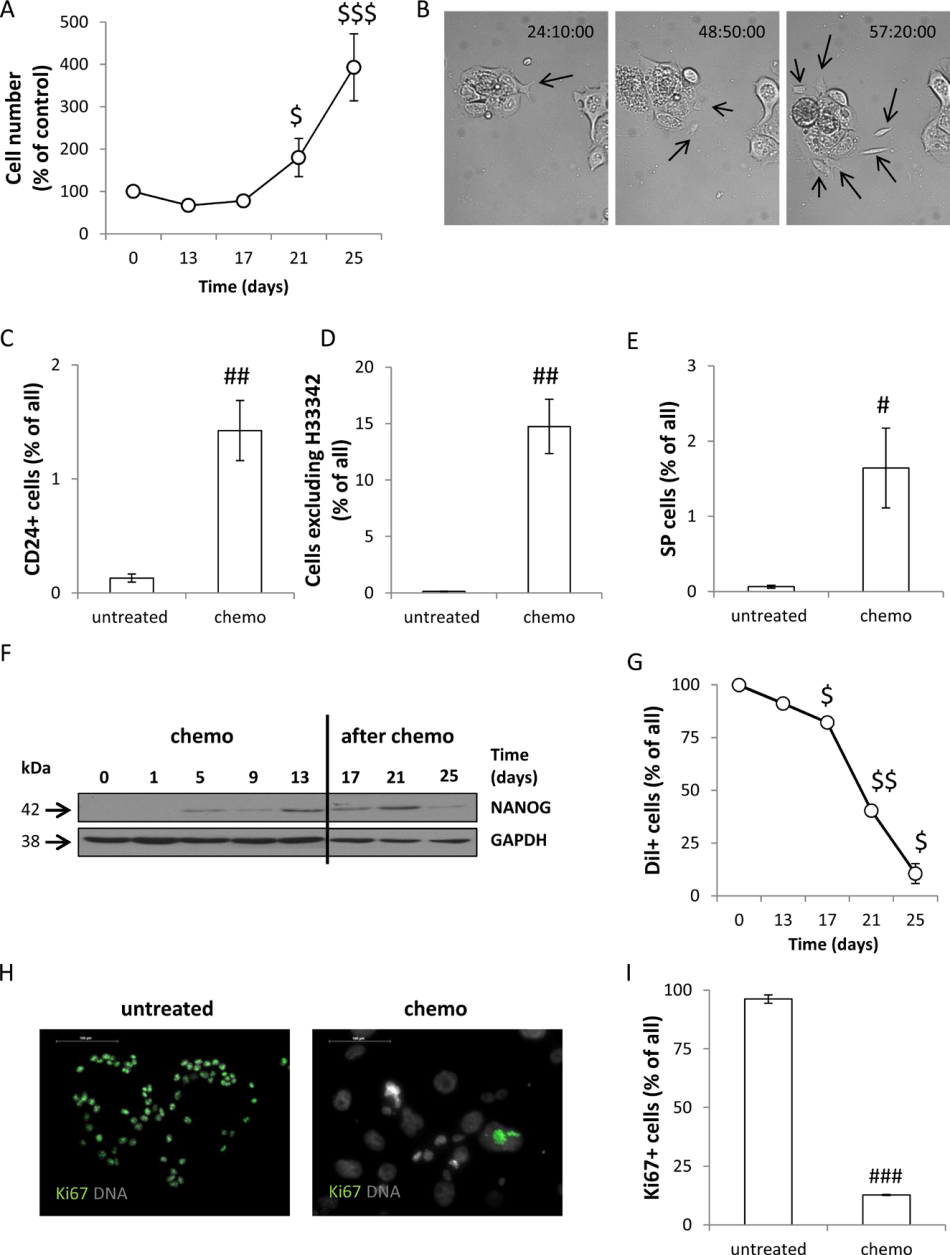
Senescent HCT116 cells exhibit certain features of cancer stem cells and re-populate *in vitro* culture after doxorubicin removal (**A**) Quantification of cell number at various time points of the AFTER CHEMO protocol. Cells were counted in Bürker's chamber. (**B**) Senescent cells produce progeny. HCT116 cells were seeded in 6-well plate and their behaviour was recorded starting from the 17th day of the AFTER CHEMO protocol. Representative photos were acquired by time-lapse imaging. Progeny originating from senescent, hypertrophic cells is indicated by black arrows. Original magnification 160×. (**C**) Evaluation of percentages of CD24^+^ cells on the 13th day (CHEMO). Cells were incubated with an anti-CD24-FITC antibody and percentages of positive cells were determined by flow cytometry. Cells labeled with an isotypic IgG were used as a negative control. (**D**) Quantification of H33342 excluding cells by flow cytometry. (**E**) Quantification of side population (SP) on the 13th day (CHEMO). Cells stained with H33342 were co-incubated with Verapamil and analyzed by flow cytometry. SP = a percentage of cells excluding H33342 in the absence of Verapamil minus a percentage of cells excluding H33342 when Verapamil is present. (**F**) Representative blot shows the expression of NANOG. GAPDH served as a loading control. (**G**) HCT116 cells persist in non-proliferating state during doxorubicin administration. Cells were stained with lipophilic, red-fluorescent dye Dil on the 1st day and the percentage of cells retaining Dil^+^ was determined with flow cytometry, at each time point. Unstained cells were used as a negative control. (**H**) Representative photos show Ki67 staining in untreated and treated cultures on the 13th day (CHEMO); immunofluorescence for Ki67-visualized as green (AlexaFluor 488), nuclei-as grey (H33342). Original magnification 400×. Data acquired by fluorescent confocal microscopy. (**I**) Automatic evaluation of a percentage of Ki67 positive cells. Each bar represents mean ± SEM, N ≥ 3; statistical significance ^#^*p* < 0.05, ^##^ *p* < 0.01, ^###^*p* < 0.001-untreated vs. CHEMO (day 13th); ^$^*p* < 0.05, ^$$^*p* < 0.01, ^$$$^*p* < 0.001 – AFTER CHEMO vs. day 13th (CHEMO).

We observed the six-fold increase in the number of cells within two weeks after doxo removal (*p* < 0.001, Figure 2A). Using a *time-lapse* technique we confirmed that senescent, hypertrophic HCT116 cells give rise to viable, migrating and proliferating progeny (Figure 2B, Supplementary Movie S1). Among the cells treated with CHEMO protocol the cells with stem-cell like properties were observed, as a fraction of CD24^+^ cells (Figure 2C) and the percentage of cells excluding H33342 (Figure 2D, Supplementary Figure S2A) increased. Only a small part of the latter subpopulation was sensitive to Verapamil, an inhibitor of ABCB1 and ABCC1 pumps (Figure 2E). These cells also expressed a stemness factor NANOG (Figure 2F). On the other hand, we found that the number of CD133^+^ (Supplementary Figure S2B) and CD44^+^ (Supplementary Figure S2C) cells as well as the ALDH activity (Supplementary Figure S2D) were reduced in treated cultures. During doxo administration HCT116 cells retained red-fluorescent, membrane dye Dil. That indicates the lack of proliferation (Figure 2G, Supplementary S2E), as in proliferating cells Dil staining is reduced with each division. Accordingly, after doxo removal the percentage of Dil^+^ cells decreased significantly, that suggests their intensive proliferation (Figure 2G, Supplementary Figure S2E). Unlike the untreated HCT116 cells, their senescent counterparts remained in non-proliferating state (Dil^+^) for many days, before they formed spheroids in matrigel (Supplementary Figure S2F). These observations are compatible with the fact that the expression of Ki67 was lost in the course of drug administration (Figure 2H, 2I). This protein is expressed in all cell cycle phases, except G0 [34]. Our results show that upon doxo treatment a majority of HCT116 cells do not enter cell cycle. Altogether, we demonstrate that the repeated doxorubicin treatment induces senescence of HCT116 cells, but drug removal leads to re-population of cell cultures. Moreover, the data presented here suggest that senescent cancer cells exhibit some features of cancer stem cells.

### A single pulse of BAF A1 transiently blocks autophagy in drug-senescent colon cancer cells

**Figure 3:**
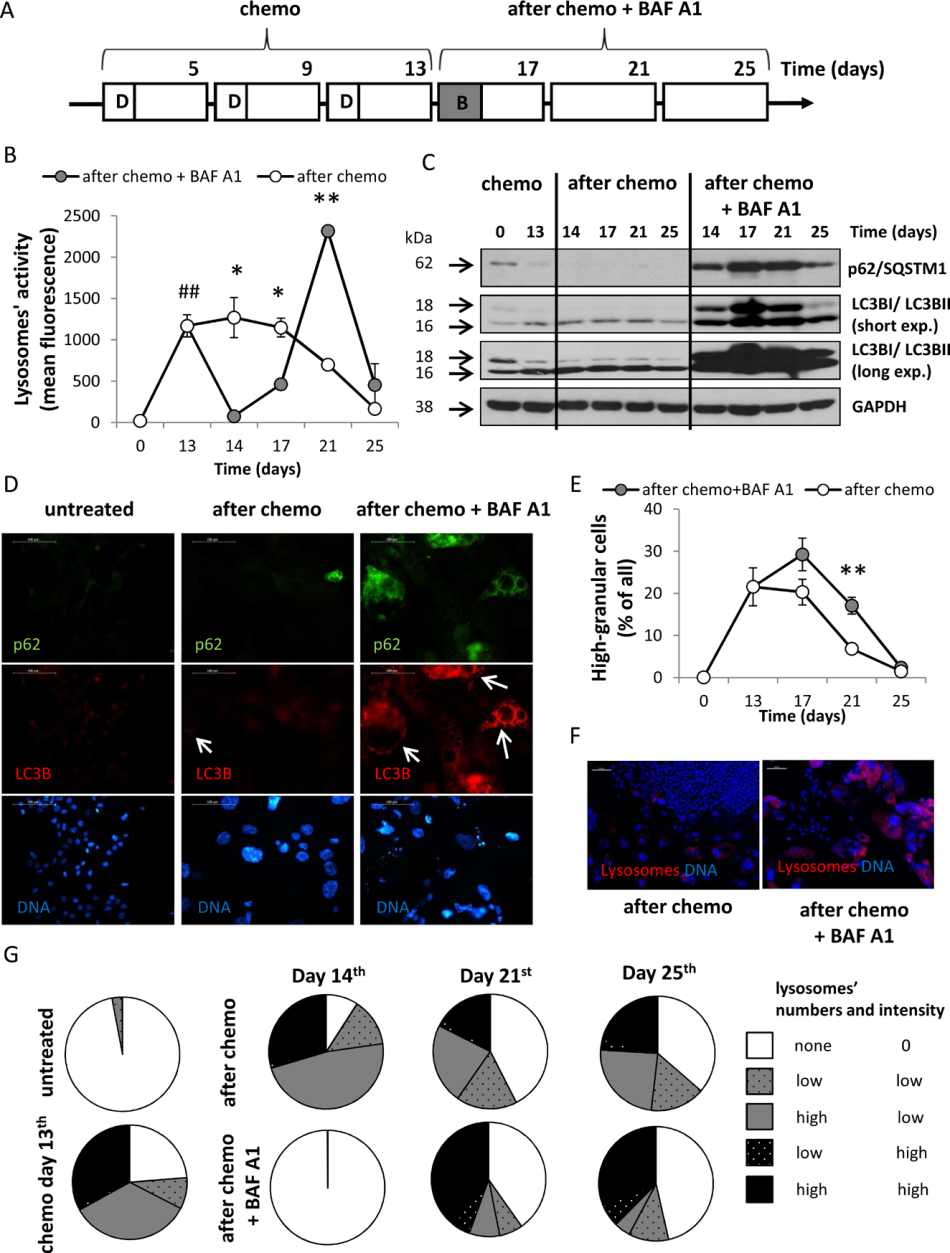
A single pulse of BAF A1 transiently blocks autophagy in senescent HCT116 cancer cells (**A**) A scheme of the experiment. After the 3rd doxorubicin cycle HCT116 cells were left untreated (AFTER CHEMO protocol) or were treated with 10 nM bafilomycin A1 (BAF A1, B) for 24 hours (AFTER CHEMO + BAF A1 protocol). Then, cells were cultured in a complete medium for the next 13 days; a medium was changed every four days. (**B**) Quantification of lysosomal activity with Lysotracker staining using flow cytometry. (**C**) Representative blots show the level of p62 and LC3BI/II proteins. GAPDH served as a loading control. (**D**) Visualization of p62 and LC3B in untreated and treated cells on the 17th day. Representative photos show LC3B staining–visualized as red (AlexaFluor 488), p62–as green (AlexaFluor 555), nuclei–as blue (H33342). Puncta of LC3BII are indicated by white arrows. Original magnification 400×. Data were acquired using fluorescence microscopy. Scale bar–100 μm. (**E**) Quantification of percentages of high-granular cells by flow cytometry using FSC/SSC analysis. (**F**) Visualization of lysosomal activity in untreated and treated cultures on the 21st day with Lysotracker–visualized as red, nuclei were stained with H33342–visualized as blue. Original magnification 100×. Data were acquired by fluorescent microscopy. Scale bar-100 μm. (**G**) Evaluation of number and intensity of lysosomes at various times after treatment. Analysis of images of cells stained with Lysotracker (stains lysosomes) and H33342 (stains nuclei). Cells were divided in terms of lysosomes number and fluorescence intensity. Each bar represents mean ± SEM, *N* ≥ 3. ^#^*p* < 0.05, ^##^*p* < 0.01, ^###^*p* < 0.001 untreated vs. CHEMO (day 13th); *p < 0.05, **p < 0.01, ***p < 0.001–AFTER CHEMO vs. AFTER CHEMO + BAF A1.

To test whether inhibition of autophagy affects division of senescent cancer cells, after the 3rd doxorubicin's cycle, HCT116 cells were treated with 10 nM BAF A1 (B) for 24 hours. The inhibitor was then removed and the cells were cultured in the drug-free medium for the next 13 days (Figure 3A, AFTER CHEMO + BAF A1 protocol). BAF A1 is an inhibitor of the late phase of autophagy, as it prevents maturation of autophagic vacuoles by inhibiting fusion between autophagosomes and lysosomes [35]. Using red-fluorescent dye *Lysotracker*, which labels acidic organelles, we show that fluorescence intensity measured by flow cytometry was significantly higher in cells treated with CHEMO protocol than in untreated cells (*p* < 0.01, Figure 3B). The fluorescence intensity was strongly reduced on the 14th day after co-incubation with BAF A1 (*p* < 0.05, Figure 3B). Furthermore, we determined the expression of two markers of autophagy - p62/SQSTM1 and LC3B. During autophagy, a cytosolic form of LC3B (LC3B-I) is conjugated to phosphatidylethanolamine to form LC3-phosphatidylethanolamine conjugate (LC3B-II), which is recruited to autophagosomal membranes. Then, LC3B-II is degraded by lysosomal hydrolases after the fusion of autophagosomes with lysosomes. p62/SQSTM1 (p62), a ubiquitin- and LC3-binding protein, is directly degraded by autophagy [35]. Using western blotting, we demonstrate that the level of p62 was reduced and LC3BII/LC3BI ratio was increased by the CHEMO protocol (Figure 3C). Moreover, BAF A1 treatment up-regulated expression of p62 and LC3B (both forms). It is noteworthy that inhibition of autophagy was maintained for at least seven days after removal of BAF A1 (Figure 3C). Using immunofluorescence we verified that a robust accumulation of p62 and LC3B occurs in BAF A1-treated cells on the 17th day. However, not all cells expressing LC3B were positive for p62. It suggests that autophagy was re-activated (Figure 3D). Likewise, lysosomal activity (measured by flow cytometry) in cells treated with the AFTER CHEMO + BAF A1 protocol was strongly enhanced on days 14 and 21. On the 21st day, these cells exhibited 3 times higher mean fluorescence intensity (*p* < 0.01, Figure 3B, 3F) and the cultures were characterized by higher number of granular cells when compared to controls (*p* < 0.01, Figure 3E). As shown in Figure 3C, the expression of p62 was reduced and LC3BII/ LC3BI ratio increased strongly on the 25th day in BAF A1-treated cells, which further confirms re-activation of autophagy. Analysis of the images showing lysosomes and DNA staining (e.g. Figure 3F) demonstrated that after BAF A1 treatment the activity of lysosomes was completely inhibited in AFTER CHEMO-treated HCT116 cells (Figure 3G). Although, a few days after BAF A1 was removed, a subpopulation of cells with the high lysosomes activity appeared (Figure 3G). Taken together these results demonstrate that a single pulse BAF A1 treatment strongly blocks autophagy in doxo-treated HCT116 cells. However, a few days later the process is re-activated, at least in a subpopulation of cancer cells.

### Senescent cancer cells are resistant to BAF A1 treatment

**Figure 4:**
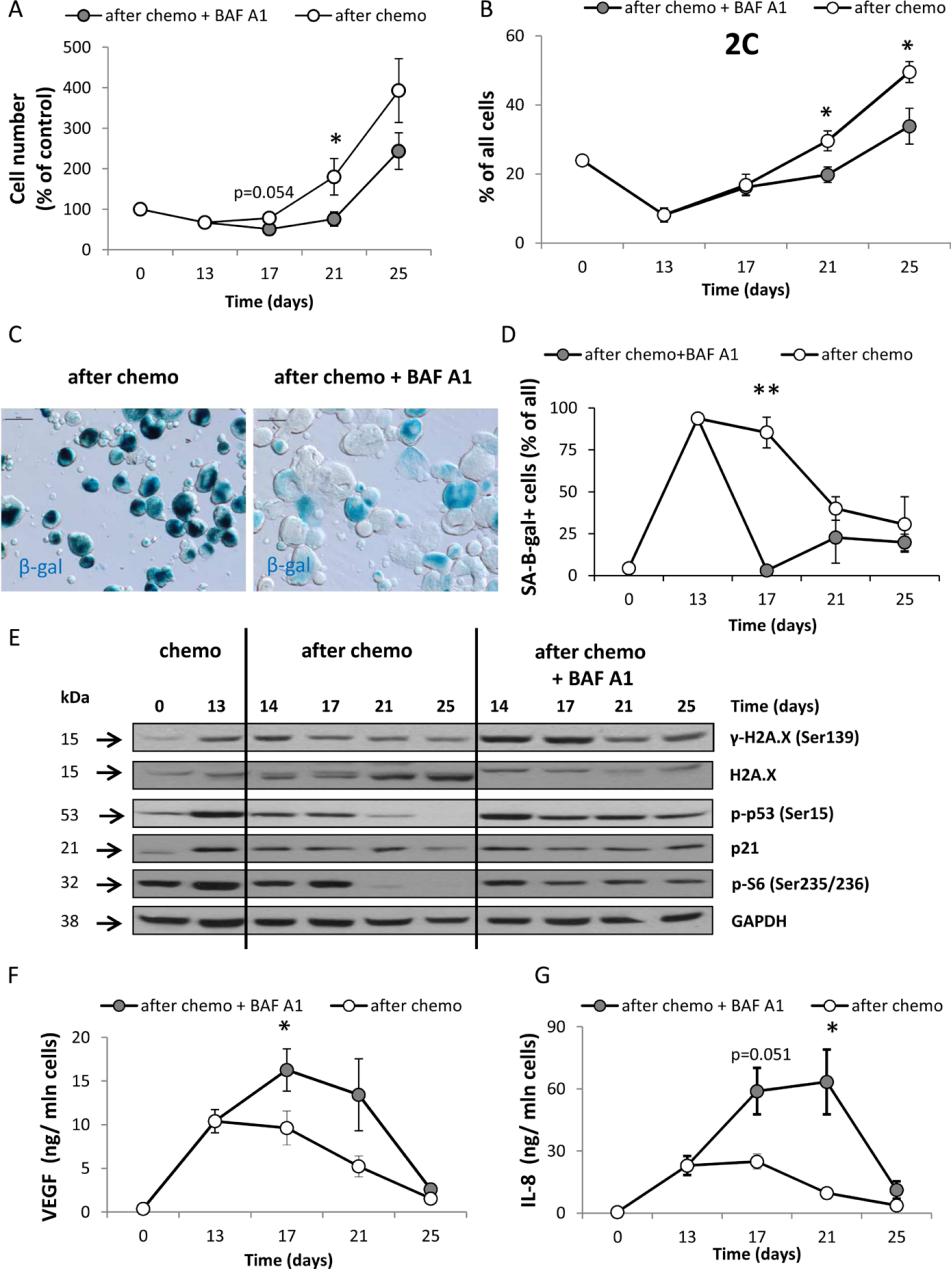
A single pulse of BAF A1 transiently delays re-population of HCT116 cell cultures (**A**) Evaluation of cell number in cultures treated with the AFTER CHEMO or the AFTER CHEMO + BAF A1 protocol at various times. Cells were counted in Bürker's chamber. (**B**) Quantification of percentages of diploid progeny entering G0/G1 phase (with 2C DNA content). Cell cycle analysis was performed using PI staining and flow cytometry. (**C**) Detection and quantification of senescent cells using SA-β-gal staining on cytospined cells. Representative photos were acquired using light microscopy on the 21st day; original magnification 200×; scale bar– 100 μm. (**D**) Quantification of SA-β-gal^+^ cells in treated cultures. (**E**) Representative blots show the levels of DDR and geroconversion proteins: γ-H2A.X, H2A.X, p-p53, p21 and p-S6. Detection of GAPDH served as a loading control. (**F**–**G**) Secretion of SASP cytokines VEGF (F) and IL-8 (G) in supernatants harvested from treated cultures was determined by colorimetric ELISA. Results were normalized to total cell number counted in Bürker's chamber. Each bar represents mean ± SEM, N ≥ 3.*p < 0.05, **p < 0.01, ***p < 0.001–AFTER CHEMO vs. AFTER CHEMO + BAF A1.

We found that re-population of HCT116 cell cultures was delayed up to 21 days after BAF A1 treatment (*p* < 0.05, Figure 4A) and the number of diploid cells entering G0/G1 phase (with 2C DNA content) was reduced (*p* < 0.05, Figure 4B). To elucidate possible mechanisms of BAF A1-induced early delay in re-population of colon cancer cell cultures, we analyzed three factors that may affect the cell number: senescence, cell death and proliferation. Although, HCT116 cells treated with the AFTER CHEMO + BAF A1 protocol exhibited morphological alterations and hypertrophy for longer time than their counterparts (day 21st, Figure 4C), SA-β-gal staining disappeared in these cultures (Figure 4C, 4D). We also found higher levels of p21, p-p53, H2A.X and p-S6 (Figure 4E) proteins and the increased production of SASP factors: VEGF (Figure 4F) and IL-8 (Figure 4G) in these cells in comparison to the controls.

**Figure 5:**
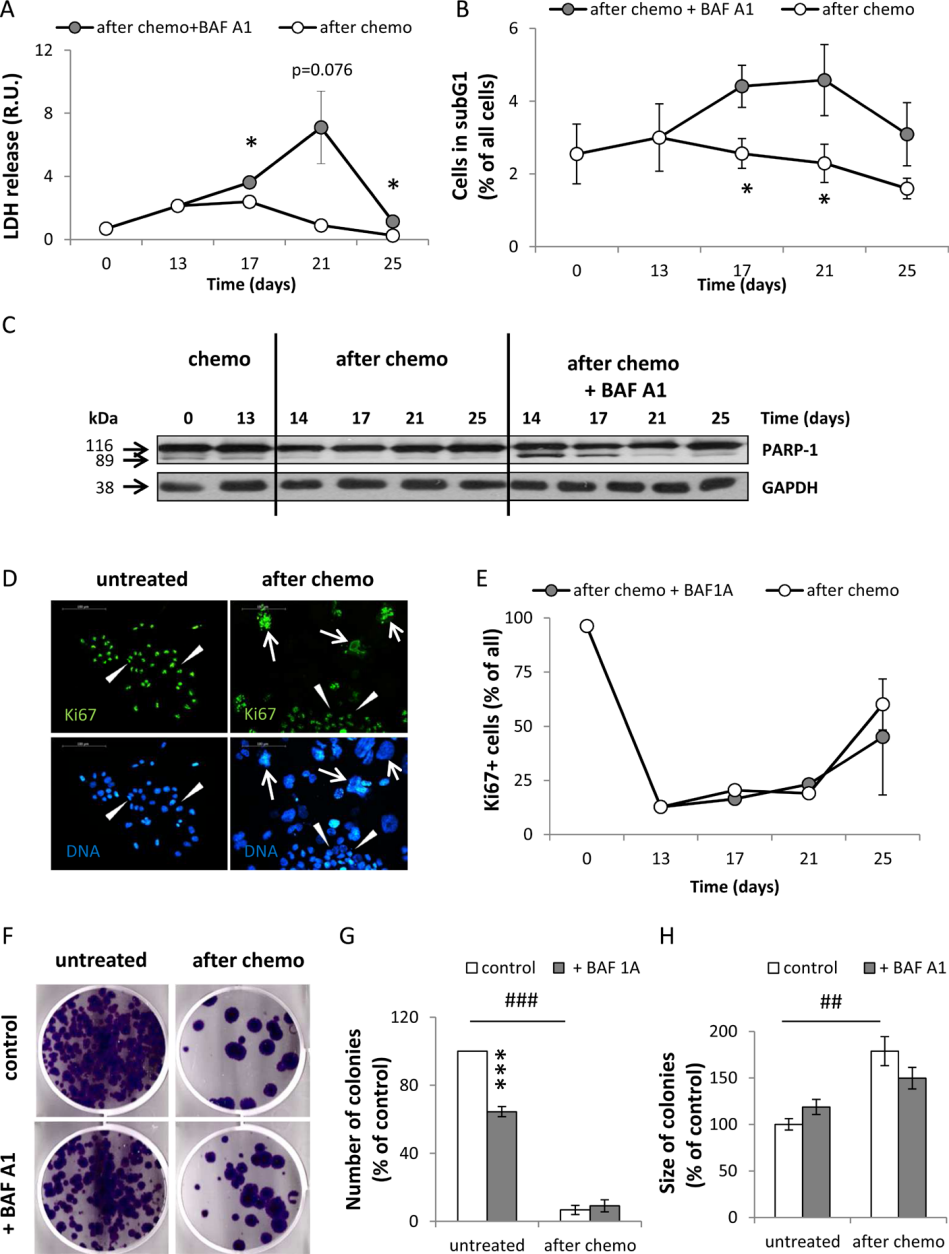
Senescent HCT116 cancer cells are resistant to BAF A1 treatment (**A**) Evaluation of cell mortality using LDH assay. Cells were treated with the AFTER CHEMO or the AFTER CHEMO + BAF A1 protocol. Results were normalized to total cell number counted in Bürker's chamber. (**B**) Quantification of percentages of cells in the subG1 phase (with DNA content < 2C). Cell cycle analysis was performed using PI staining and flow cytometry. (**C**) Representative blot shows the level of PARP-1. GAPDH served as a loading control. (**D**) Representative photos show Ki67 staining in untreated and HCT116 cells treated with the AFTER CHEMO protocol on the 21st day: Ki67 visualized as green (AlexaFluor 488), and nuclei visualized as blue (H33342). Ki67^+^ polyploid cells are indicated by white arrows, Ki67^+^ progeny or untreated HCT116 cells indicated by white arrowheads. Original magnification 200×. Data obtained by fluorescent microscopy. Scale bar–100 μm. (**E**) Automatic evaluation of percentage of Ki67 positive cells. (**F**) Visualization of colonies formed by untreated and doxo-treated cells co-incubated with BAF A1. Doxo-treated cells (1 000 cells/6-well plate) were harvested and seeded on the 14th day just after BAF A1 removal. Medium was replaced every week. Representative photos were taken three weeks after seeding. (**G**). Evaluation of numbers of colonies formed by untreated or doxo-treated cells co-incubated with BAF A1. (**H**) Evaluation of size of colonies formed by untreated or doxo-treated cells co-incubated with BAF A1. Each bar represents mean ± SEM, N ≥ 3.*p < 0.05, **p < 0.01, ***p < 0.001 –AFTER CHEMO vs. AFTER CHEMO + BAF A1, ^#^*p* < 0.05, ^##^ *p* < 0.01, ^###^*p* < 0.001 - untreated vs. AFTER CHEMO.

Activation of H2A.X and p53, as well as an increased IL-8 production, could be indicators of cell death. Indeed, using a LDH release test we demonstrated that HCT116 cells treated with the AFTER CHEMO+BAF A1 protocol exhibited higher mortality than their counterparts (Figure 5A). This effect was preceded by the increase in a proportion of cells in the subG1 phase (with DNA content < 2C) (Figure 5B). We corroborated these data by detecting the cleaved PARP-1 using western blotting. On the days 14 and 17 BAF A1-treated senescent cells had the increased level of cleaved PARP-1 than their counterparts (Figure 5C). This effect was not observed at other time points.

It should be noted that BAF A1 treatment did not affect the percentage of Ki67 positive cells (Figure 5E). Ki67 was expressed not only in progeny, but also in some polyploid cells (Figure 5D). We show that approximately 90% of CHEMO-treated HCT116 cells are in G0 phase (Figure 5E). Senescent cells have been reported to be blocked in G1 or G2 phase of the cell cycle [36]. Therefore, it was not clear whether the presence of Ki67 indicated cells, which were actively cycling or halted in one of the cell cycle phases. We studied the effect of BAF A1 treatment on clonogenic potential of individual doxo-treated cells, by seeding them at low density (1000 cells per well) on the 14th day and examining their ability to form colonies three weeks later. AFTER CHEMO-treated HCT116 produced significantly fewer colonies than untreated cells (Figure 5F, 5G), although these colonies were bigger (Figure 5F, 5H). There was no significant difference in the numbers of colonies derived from senescent cells and their counterparts treated with BAF A1 (Figure 5F, 5G). In contrast, BAF A1 treatment decreased significantly the number of colonies derived from untreated HCT116 (Figure 5F, 5G). We performed a detailed cell cycle analysis using PI staining (Supplementary Figure S3) to check, if BAF A1 affects predominantly other than senescent cells. Indeed, upon BAF treatment the proportion of diploid cells (with DNA content ≤ 4C) decreased(Figure 4B, Supplementary Figure S3B, S3C), whereas proportion of polyploid cells, especially with DNA content > 8C, increased (Supplementary Figure S3F, S3G, S3H, S3I).

To verify our observations, we treated human glioblastoma LN18 cells with repeated cycles of 50 nM doxo. LN18 cells displayed the signs of senescence already after the 1st cycle of the treatment and, in contrast to HCT116 cells, started to proliferate afterwards. Therefore, at the moment of BAF A1 treatment only 20% of CHEMO-treated LN18 cells showed features of senescence, e.g. SA-β-gal positivity (Supplementary Figure S4A). BAF A1 treatment (consistently with autophagy inhibition) increased the level of p62 protein and changed the proportion of LC3B II/I (Supplementary Figure S4D), but the effects were not as prominent as in HCT116 (Figure 3C). LN18 cells treated with the AFTER CHEMO + BAF A1 protocol exhibited the increased granularity (Supplementary Figure S4B) and hypertrophy (Supplementary Figure S4C), augmented p-p53, γ-H2A.X, and p-S6 expression (Supplementary Figure S4D), as wells as up-regulated secretion of VEGF (Supplementary Figure S4E) and IL-8 (Supplementary Figure S4F) in comparison to the control group. Moreover, these cells showed the increased mortality (Supplementary Figure S4G), reduced proportion of diploid cells with 2C DNA content and the enriched proportion of cells with DNA content higher than 2C, but lower than 16C (Supplementary Figure S5). A single pulse of BAF A1 produced more pronounced effects on re-population of glioblastoma cultures (Supplementary Figure S4H) than among colon cancer cells counterparts (Figure 4A). It likely reflects the difference in the proportion of senescent cells at the time of BAF A1 application.

Subsequently we studied if the effects of BAF A1 treatment depend on a mode of action of chemoterapeutic agents that are used in colon cancer patients. HCT116 cells treated with another senescence-inducing agent irinotecan (irino) (Supplementary Figure S6A) were resistant to BAF A1 treatment (Supplementary Figure S6B). In contrast, the cells treated with oxaliplatin (oxa), which does not induce senescence (Supplementary Figure S6A), showed moderate sensitivity towards BAF A1 (Supplementary Figure S6B). We observed that the cells exhibited strong induction of autophagy upon irino treatment. The level of p62 was down-regulated, whereas the LC3BII/ I ratio was increased (Supplementary Figure S6C). The opposite effects were noticed after oxa treatment (Supplementary Figure S6C). Taken together, these data demonstrate that, in contrast to non-senescent cancer cells, senescent cells are resistant to BAF A1 treatment.

### A single pulse BAF A1 treatment transiently affects some markers of cancer stem cells

**Figure 6:**
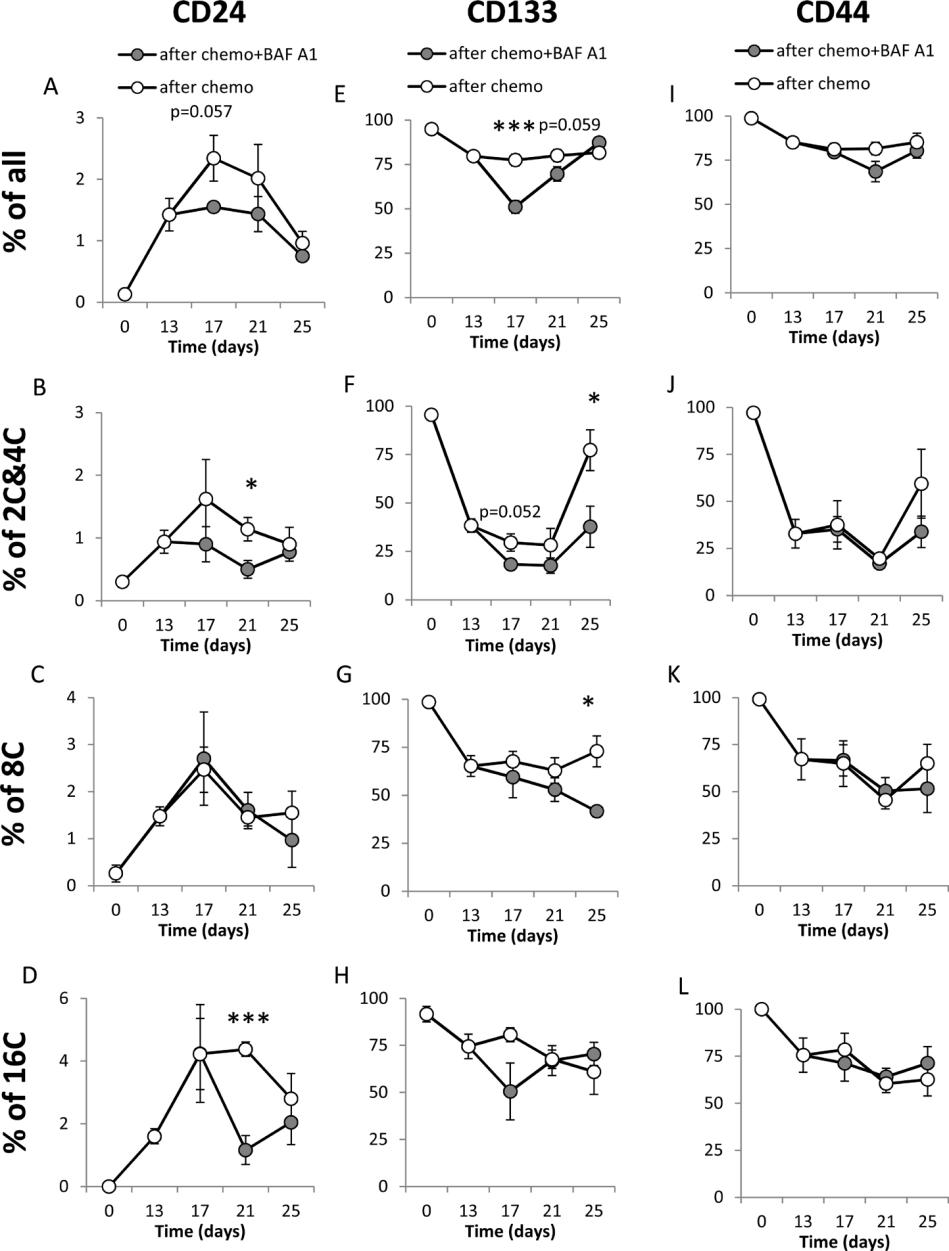
A single pulse of BAF A1 transiently affects stemness markers in senescent HCT116 cells (**A**–**D**) Evaluation of percentage of CD24 positive cells in cultures treated with the AFTER CHEMO or the AFTER CHEMO + BAF A1 protocol at various time points. Cells stained with H33342, as a ploidy discriminator, were probed with an anti-CD24-FITC antibody. Percentages of all cells (A), and CD24^+^ cells with 2C&4C (B), 8C (C) and 16C (D) DNA content were determined by flow cytometry. Cells labeled with isotypic IgGs were used as a negative control. (**E**–**H**) Evaluation of percentage of CD133^+^ cells at various time points. Cells stained with H33342 were probed with an anti-CD133-APC antibody. Percentages of all cells (E), and CD133^+^ cells with: 2C&4C (F), 8C (G) and 16C (H) DNA content were determined by flow cytometry. (**I**–**L**) Evaluation of percentages of CD44^+^ cells at various time points. Cells stained with H33342, were incubated with an anti-CD44-AlexaFluor700 antibody. Percentages of all cells (I), and CD44^+^ cells with: 2C&4C (J), 8C (K) and 16C (L) DNA content were determined by flow cytometry. Each bar represents mean ± SEM, *N* ≥ 3.*p < 0.05, **p < 0.01, ***p < 0.001 – AFTER CHEMO vs. AFTER CHEMO + BAF A1.

Next we tested whether BAF A1 treatment affects the expression of cancer stem cell markers and their functions. As shown in the Figure 6A, CD24 was expressed on the higher number of CHEMO-treated HCT116 cells than their counterparts (*p* < 0.01). Following chemotherapy, the percentage of CD24^+^ cells increased further till the 17th day and subsequently decreased (Figure 6A). BAF A1 reduced the number of CD24^+^ cells in a whole population (*p* = 0.057, Figure 6A). Using multicolor staining with H33342 as a ploidy discriminator, we showed that on the 21^st^ day BAF A1 treatment resulted in the significant reduction in the number of CD24^+^ cells among diploid cells (*p* < 0.05, Figure 6B) and the cells with 16C DNA content (*p* < 0.001, Figure 6D).

**Figure 7:**
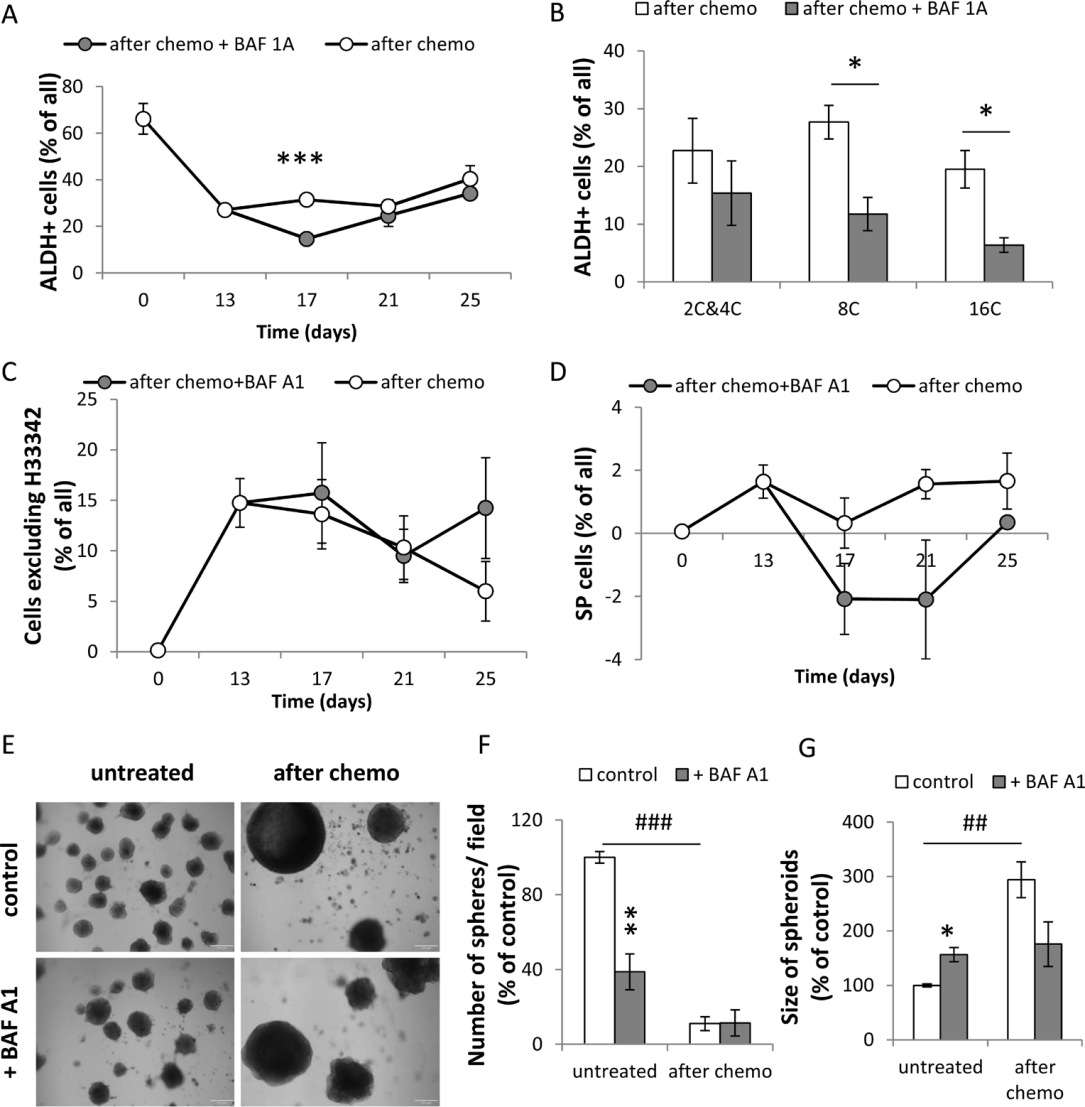
A single pulse of BAF A1 transiently impairs ALDH activity and H33342 exclusion in senescent HCT116 cells (**A**) Evaluation of percentages of HCT116 cells treated with the AFTER CHEMO protocol or the AFTER CHEMO + BAF A1 protocol showing ALDH activity at various time points. Cells co-incubated with DEAB, an ALDH inhibitor, served as a negative control. (**B**) Evaluation of percentage of HCT116 cells showing ALDH activity on the 17th day. Cells stained with H33342, as a ploidy discriminator, were stained for an ALDH activity. Percentage of cells with: 2C&4C, 8C and 16C DNA content displaying ALDH activity were determined by flow cytometry. (**C**) Quantification of cells excluding H33342 by flow cytometry. (**D**) Quantification of side population (SP). SP = a percentage of cells excluding H33342 in the absence of Verapamil minus a percentage of cells excluding H33342 in the presence of Verapamil. Cells stained with H33342 were co-incubated with Verapamil and analyzed by flow cytometry. (**E**) Visualization of spheroids in matrigel formed by untreated and doxo-treated cells co-incubated with BAF A1. Doxo-treated cells (1 000 cells) were seeded on the 14th day just after BAF A1 removal onto 96-well plate. Media were replaced every week. Representative photos were taken three weeks after seeding. Scale bar–100 μm. (**F**) Evaluation of the number of spheroids formed by untreated or doxo-treated cells co-incubated with BAF A1. (**G**) Evaluation of size of spheroids formed by untreated or doxo-treated cells co-incubated with BAF A1. Spheroids in the middle of well (hot spot) were calculated. Each bar represents mean ± SEM, *N* ≥ 3.*p < 0.05, **p < 0.01, ***p < 0.001–AFTER CHEMO vs. AFTER CHEMO + BAF A1.

Almost 100% of untreated HCT116 cells and ∼80% of CHEMO-treated HCT116 expressed CD133 (Figure 6E) and CD44 antigens (Figure 6I). After doxo removal the percentages of CD44+ and CD133+ cells among diploid cells returned to high basal levels (Figure 6F, 6J), in contrast to their reduction among polyploid cells (Figure 6G, 6H, 6K, 6L) We demonstrate that BAF A1 treatment diminished the number of CD133^+^ cells (*p* < 0.001, Figure 6E) and CD44^+^ cells (*p* = 0.07, Figure 6I). This short-term reduction in the number of CD133^+^ corresponded mostly to diploid cells (*p* = 0.052, Figure 6F). As shown in the Figure 7A, the higher number of untreated HCT116 cells exhibited ALDH activity than CHEMO-treated cells (*p* < 0.01). BAF A1 treatment led to the significant reduction in the percentage of cells with ALDH activity on the 17th day (*p* < 0.001, Figure 7A), but only in polyploid cells (Figure 7B). Furthermore, we demonstrated that a subpopulation of cells excluding H33342 increased significantly after drug treatment (*p* < 0.01), while BAF A1 had no effect (Figure 7C). On the day 17th BAF A1 treated cells displayed the slight, but not significant decrease in the proportion of cells excluding H33342, that were sensitive to Verapamil (Figure 7D).

Last but not least, we studied whether BAF A1 treatment can affect spheroid-forming capacity of doxo-treated cells. Therefore, on the 14th day we seeded 1000 cells per matrigel-filled well in 96-well plate and examined them for spheroid formation three weeks later. In contrast to non-senescent HCT116 cells, BAF A1 did not change the numbers of spheroids derived from TIS cells (Figure 7E, 7F). Spheroids formed by the doxo-treated colon cancer cells were larger than those formed by the untreated cells (Figure 7E, 7G). To summarize, these data show that BAF A1 affects differently polyploid or diploid cancer cells with stem cell-like properties.

### A single pulse of BAF A1 in doxo-treated cancer HCT116 cells reactivates their ability to form tumors in NOD/SCID mice

**Figure 8:**
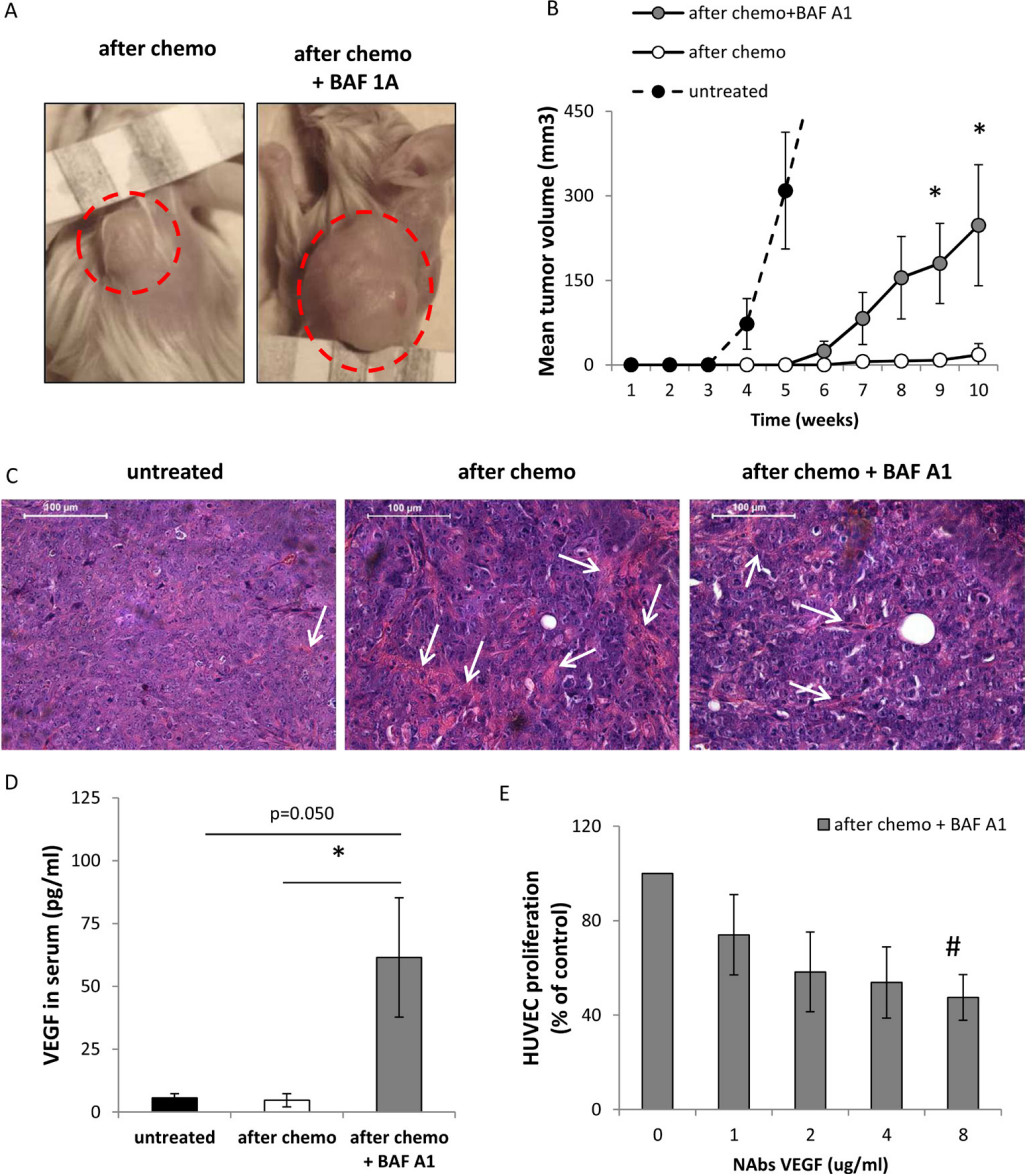
Pulse BAF A1 treatment of senescent HCT116 cells accelerates tumor growth in NOD/SCID mice Cells treated with the AFTER CHEMO or the AFTER CHEMO + BAF A1 protocol for 14 days were trypsinized, counted and injected in the back of NOD/SCID mice (5 000 cells in 100 μl matrigel mixed with PBS, 1:1); *n* = 7. Untreated HCT116 cells were used as controls, *n* = 6. (**A**) Representative photos show tumors (indicated by red dotted circles) at the end of the experiment. In AFTER CHEMO group, the tumor grew in 1/7 mice. (**B**) Quantification of mean volume of tumors according to formula: *V* = *D* × *d*^2^ × 0.5 (*V* is the tumor volume, *D* is the biggest dimension, *d* is the smallest dimension). (**C**) Histological evaluation of tumors. H&E staining was performed on paraffin-embedded sections. Representative photos show tumor morphology with collagen fibers (pink strands) indicated by white arrows. Original magnification 400x. Data acquired by light microscopy. Scale bar–100 μm. (**D**) Evaluation of human VEGF in sera of NOD/SCID mice by colorimetric ELISA specific for detection of human proteins. (**E**) Proliferation of HUVEC cells in response to conditioned media harvested from HCT116 cells treated with the AFTER CHEMO + BAF A1 protocol. Media were pre-incubated with an isotypic or a VEGF-neutralizing antibody (NAbs-VEGF) and proliferation of HUVEC cells using BrdU colorimetric assay was performed 48 hrs later. Each bar represents mean ± SEM. *N* ≥ 3. *p < 0.05, **p < 0.01, ***p < 0.001–AFTER CHEMO vs. AFTER CHEMO + BAF A1.

To study whether BAF A1 treatment of doxo-treated HCT116 cells affects their ability to form tumors *in vivo*, they were implanted subcutaneously in the back of 6-week old NOD/SCID mice. Cells were treated with the AFTER CHEMO or AFTER CHEMO + BAF A1 protocol for 14 days. Subsequently, cells were harvested, mixed with matrigel and 5 000 cells per mice were injected. Tumors were allowed to grow for 10 weeks and tumor sizes were measured once per week. Untreated HCT116 cells served as controls. Cells treated with the AFTER CHEMO+ BAF A1 protocol started to form tumors four weeks earlier than their counterparts and tumors developed in 5 per 7 animals. Tumor grew only in 1 out of 7 animals inoculated with the AFTER CHEMO-treated cells (Figure 8A, 8B). As a result, at the end point the AFTER CHEMO+ BAF A1-treated cells produced bigger tumors in comparison to controls (*p* < 0.05, Figure 8B). Morphological analysis revealed that senescent HCT116-derived tumors displayed higher density of collagen fibers and better vascularization than their counterparts as evidenced by hemotoxylin/eosin staining (Figure 8C).

To elucidate the mechanism of facilitated tumor growth in NOD/SCID mice, we determined the production of human VEGF in sera of tumor-bearing mice. Cells treated with the AFTER CHEMO + BAF A1 protocol secreted more VEGF *in vitro* that their counterparts (Figure 4A). VEGF is a major pro-angiogenic factor, which supports endothelial cell proliferation and tumor neoangiogenesis [37]. Additionally, this growth factor was shown to assist both endothelial [38] and tumor cells to escape senescence [39, 40]. Here, we demonstrate that the levels of VEGF, estimated by ELISA (specific for human protein) were higher in sera of mice injected with BAF A1-treated senescent cells than in sera of animals injected with their counterparts (Figure 8D). To check, whether VEGF released by cancer cells might affect endothelial cell proliferation, we incubated HUVEC cells with conditioned media harvested from HCT116 cells treated with the AFTER CHEMO + BAF A1 protocol in the presence of an isotypic or VEGF-neutralizing antibody (NAbs-VEGF). Co-incubation with NAbs-VEGF decreased proliferation of HUVEC (Figure 8E). Taken together, these data suggest that a pulse BAF A1 treatment increases tumorigenic potential of senescent HCT116 and, consequently, the efficiency of tumor formation in NOD/SCID animals.

## DISCUSSION

### Senescent cells display several features of cancer stem cells

Clinical observations indicate the appearance of senescent cells following therapy [8–10]. *In vitro* studies demonstrate the escape of cancer cells from senescence [16–18, 41–45], which taken together suggest poor prognosis due to cancer re-growth [8–10, 14]. This phenomenon has not been satisfactorily explained. Cancer cell senescence has been modeled using HCT116 cells treated with a single dose of doxorubicin [18, 41, 46, 47]. We treated HCT116 colon cancer cells with three doxorubicin cycles to obtain more stable and homogenous population of senescent cells [48] and to mimic processes occurring in cancer patients subjected to repeated chemotherapy cycles over long time. We demonstrate that in response to the treatment, a majority of HCT116 cells showed features of senescence exemplified by flatten morphology and increased size, growth arrest, polyploidy, augmented SA-β-Gal and SASP. After doxorubicin removal, the number of cells significantly increased, which was associated with atypical divisions of the polyploid cells. Previously, we had showed an indirect evidence that some doxorubicin-treated HCT116 cells can give rise to progeny [18, 41]. Rajaraman and coworkers proposed that polyploid cells undergo neosis, an amitotic type of cell division, followed by asymmetric intracellular cytokinesis that produces small cells with stem-cell-like characteristics [16, 19, 49]. Generation of small cells (budding) was described in tumor cell lines of different tissue origins [17, 43, 50]. Accumulation of the nuclear markers of stemness: OCT4, NANOG and SOX2 in rare, highly polyploid cells was also reported [17, 51]. Accordingly, we demonstrate that senescent HCT116 cells possess several properties of cancer stem cells, namely: elevated NANOG expression, increased Hoechst 33342 exclusion, increased CD24 expression, an ability to divide asymmetrically, clonogenic and spheroid-forming potential. Furthermore, using Ki67 staining and Dil retention assay, we show that during doxo administration, a majority of HCT116 cells are maintained in G0 phase. Accordingly, Zhang and coworkers showed that polyploid cancer cells express normal and cancer stem cell markers, divide asymmetrically and cycle slowly [42]. Erenpreisa's group reported that polyploid cells expressing stemness (OCT4, SOX2, NANOG) and senescence (p16) markers in samples from tumors, that were non-responsive to neoadjuvant chemotherapy, were Ki67 and CD44 positive [52]. In contrast, Zhang and colleagues documented that SA-β-Gal-negative polyploid cancer cells can divide by budding and give rise to cancer stem cells [43].

To clarify whether polyploid cells or their progeny exhibit cancer stem cell-like properties, we analyzed the numbers of CD24, CD44 and CD133 positive cells, using H33342 staining as a ploidy discriminator. We show that upon doxo treatment the percentage of CD24^+^ cells increased, especially among polyploid cells. Likewise, the numbers of CD133^+^ and CD44^+^ cells decreased. Interestingly, after doxo removal the percentages of CD44^+^ and CD133^+^ cells among diploid cells returned to high basal levels, in contrast to their reduction among polyploid cells. These data suggest that doxo-treated colon cancer cells exhibit dynamic changes in CD24, CD133 and CD44 antigen presentation, which may have the impact on e.g. the efficacy of CSC-targeted immunotherapy. The distribution of CD44^+^/CD24^+^ cells in colorectal cancer is under dispute, although it has been demonstrated that between 50 and 68% of patients suffering from colorectal cancers have high level of CD24^+^ cells [53]. Recent studies demonstrated that CD44^+^/CD24^+^ colorectal cancer cells show greater clonogenic potential *in vitro* and tumor initiation *in vivo* [54]. Du et al. indicated that the HCT116 cells with the high expression of CD44 and CD133 showed tumor-initiating capability [55]. On the other hand, it was reported that both CD133^+^ and CD133^-^ subpopulations formed colonospheres in *in vitro* cultures and were capable of long-term tumorigenesis in NOD/SCID mice. However, during the metastatic transition, CD133^+^ cells might give rise to the more aggressive CD133^-^ subset [56]. Therefore, our results are in line with those notions pointing to variability in CSC phenotypes, potential of non-CSCs to switch to CSC-like cells and the existence of dynamic balance of CSCs [21]. Altogether, we conclude that doxo-induced senescent cells display a specific phenotype, being a mixture of cancer stem-like and differentiated cell features. Nonetheless, links between senescence and stemness require further elucidation at a single cell level.

### Senescent cells are resistant to autophagy inhibition

Non-dividing cells cannot redistribute and eliminate damaged organelles, proteins or aggregates through cell division [25], which implies the importance of autophagy for senescent cells [57]. This process was demonstrated to promote colon cancer cell survival by preventing cell death, induced by radio- and chemotherapy [58–61]. The results of clinical studies combining autophagy inhibitor HCQ with chemotherapy have been already published [33], leaving several issues unclear, in particular, the impact of TIS on the acquired drug resistance. Here, we show that doxo-treated colon cancer HCT116 cells exposed to a single pulse of BAF A1 (a specific inhibitor of lysosomal acidification, similar to HCQ and used widely as an autophagy inhibitor), reduced their proliferation at early stages. Importantly, the effect was associated with the decrease in the number of diploid cells, but the augmented numbers of polyploid ones. Nonetheless, at later stages of experiment, the proliferation rate increased and autophagy was re-activated, at least in a subpopulation of TIS cells. The effects of a single pulse of BAF A1 on re-population of doxo-treated glioblastoma LN18 cultures were more pronounced than in colon cancer cells, and were correlated with the lower proportion of TIS cells at the time of BAF A1 application. Additionally, we demonstrate that BAF A1 impaired colony- and spheroid-formation in the control, but not in TIS cells. We hypothesized that some of polyploid cells with re-activated autophagy produce progeny. Accordingly, autophagy/lysosomal pathway was reported to participate in chromatin processing in senescent cells [62] and segregation of functional subnuclei during their amitotic divisions [17].

Consistently, treatments with two drugs that are used in colon cancer patients – irinotecan (a senescence-inducing agent) and oxaliplatin (which does not activate senescence) produced different effects. It should be noted that doxorubicin and irinotecan activate senescence and autophagy as well. Therefore, we proposed that elevated autophagy in senescent cancer cells contributes to their high resistance to BAF A1 treatment. Interestingly, TIS lymphomas, unlike senescence models that lack a strong SASP response, were more sensitive to blockade of glucose utilization or autophagy, which led to their selective elimination through caspase-12- and caspase-3-mediated endoplasmic-reticulum-related apoptosis [63]. Polyploid cells were shown to have distinct advantage over diploid cancer cells in dealing with stress and reproduction [64].

Moreover, we demonstrate that BAF A1-treated TIS HCT116 cells, when injected to NOD/SCID mice, form tumors more effectively than TIS HCT116 cells. We hypothesize that it could be due to increased proliferation of a subpopulation of HCT116 cells that re-activated autophagy after a pulse of BAF A1 treatment and/or to some factors released by BAF A1-treated senescent colon cancer cells that might affect their interactions with stroma. Using ELISA specific for human proteins, we found significantly more VEGF in sera of mice injected with BAF-A1 treated senescent cells than in sera of animals inoculated with their counterparts. BAF A1 treatment also up-regulated VEGF secretion by senescent cells *in vitro*. We show that proliferation of HUVEC cells treated with conditioned media harvested from BAF A1-treated TIS HCT116 was enhanced, whereas treatment with a neutralizing antibody NAbs-VEGF decreased their proliferation. VEGF is a key regulator of angiogenesis [37] and is crucial for angiogenic switch during early stages of tumorigenesis [65]. VEGF is produced by normal senescent fibroblasts and enhances angiogenesis in tumors [66]. Importantly, VEGF may induce escape from senescence in endothelial cells [38] as well in colorectal cancer [39] and glioblastoma [40]. Therefore, our data suggest that TIS cancer cells are more resistant to BAF A1 treatment than untreated ones. The single pulse autophagy inhibition in senescent HCT116 cells might accelerate tumor growth in mice through SASP-mediated mechanism.

### SA-β-gal is not a specific senescence marker

We show that despite being enlarged and growth arrested, the senescent HCT116 cells treated with BAF A1 almost completely lost SA-β-gal staining. It raises a question, which markers should be used to characterize senescence. SA-β-gal activity is considered a major senescent marker [3, 67, 68] and the first choice in *in vivo* studies [13, 14, 47]. The actual involvement of this enzyme in the senescence response has not been directly demonstrated [69, 70]. SA-β-gal staining appears also in dense cell cultures that are not senescent. Therefore, we propose that measurement of DNA content that is directly related to cellular hypertrophy [7] could be more reliable marker of senescence than SA-β-gal activity. In the present study, we employed two methods to measure DNA content, both in suspension and *in situ*. Staining with Ruby [14] or vital H33342 as ploidy discriminators, allows to connect the expression of various extra- and intracellular proteins with ploidy status in alive cells by means of flow cytometry. In turn, quantification of nuclei areas [43] and their co-localization with proteins of interest in microscopic images enables to study polyploid cells in specimen. Detection of triploidy in the DNA histogram of diagnostic biopsy was proposed to be useful for decision on the expediency of chemotherapy [52].

In conclusion, we demonstrate that senescent cancer cells that appear after chemiotherapeutics exhibit certain features of cancer stem cells and may persist in a dormant state before re-establishing cancer after ending chemotherapy. Importantly, a pulse autophagy inhibition by BAF A1 in senescent cells may reactivate their tumor-forming capacities and facilitate tumor growth through SASP-mediated mechanism. Therefore, further detailed studies of mechanisms underlying TIS-related cancer maintenance and re-population are urgently needed.

## MATERIALS AND METHODS

### Chemicals and antibodies

Unless otherwise specified, chemicals and reagents were purchased from Sigma Aldrich. Antibodies against: p53 (DO-1) and p21Cip1 (C-19) were purchased from Santa Cruz Biotechnology, Ki-67 from Dako, PARP-1 from Enzo, p-p53 (Ser15), p-S6 (Ser235/236), and Nanog from Cell Signaling, p62 from Transduction Laboratories^TM^, γ-H2A.X (Ser 139) from Abcam, H2A.X and GAPDH from Millipore. Secondary anti-mouse and anti-rabbit antibodies conjugated with HRP were obtained from Vector Laboratories, and ECL reagents from Thermo Scientific. LysoTracker^®^ Red DND-99, DilC_12_(3) (Dil), secondary antibodies conjugated with AlexaFluor 488 or AlexaFluor 555 were purchased from Molecular Probes. Mounting medium, protease inhibitor cocktail and phosphatase inhibitor cocktail were obtained from Roche Diagnostics. 7-AAD, Matrigel Matrix, Matrigel Matrix Growth Factor Reduced, FITC mouse anti-human CD24, FITC mouse IgG2a, κ isotype control, AlexaFluor^®^ 700 mouse IgG2b, κ isotype control, Alexa Fluor^®^700 mouse anti-human CD44 were obtained from BD Pharmingen^TM^, APC mouse IgG1 isotype control, APC mouse anti-human CD133/1 (AC133) were purchased from Miltenyi Biotec. ELISA kits for human vascular endothelial growth factor (VEGF), and human interleukin-8 (IL-8), neutralizing antibodies against human VEGF and corresponding isotype control antibodies were procured from R&D Systems.

### Cells and treatment

Human colon HCT116 cancer cell line was kindly provided by Dr. Bert Vogelstein (Johns Hopkins University, Baltimore, MD). Authentication of cell lines was performed by Cell Line Authentication IdentiCell STR. Cells were grown under standard conditions (37°C, 5% CO_2_) in McCoy's medium supplemented with 10% fetal bovine serum, 100 units/mL of penicillin, 100 μg/mL of streptomycin, and 25 μg/mL of amphotericin B (Antibiotic-Antimycotic).

Human glioblastoma LN18 cell line (derived from a glioblastoma multiforme, WHO grade IV) was from ATCC. Cells were grown under standard conditions (37°C, 5% CO_2_) in low glucose (1 mg/ml) Dulbecco's Modified Eagle Medium (DMEM) supplemented with 10% fetal bovine serum, 100 units/mL of penicillin, 100 μg/mL of streptomycin, and 25 μg/mL of amphotericin B (Antibiotic-Antimycotic).

To induce senescence cancer cells were seeded at a density of 10 000/cm^2^ 24 hours before chemotherapeutics treatment. Next, HCT116 cells were cultured in the presence of 100 nM doxorubicin (doxo), 5 μM oxaliplatin (oxa) or 2.5 μM irinotecan (irino). LN18 cells were cultured in the presence of 50 nM doxo. This procedure was repeated three times (CHEMO protocol). Afterwards, cells were cultured in drug-free medium for two weeks. Medium was changed every four days (AFTER CHEMO protocol).

To inhibit autophagy in senescent cells, HCT116 or LN18 cells treated with the CHEMO protocol were cultured in the presence of 10 nM bafilomycin (BAF A1) for 24 h. Afterwards, senescent cancer cells were cultured in drug-free medium for two weeks. Medium was changed every four days (AFTER CHEMO + BAF A1 protocol).

Human umbilical vein endothelial cells EA.hy926 was kindly provided by Dr. Antoni Wrzosek (Nencki Institute of Experimental Biology, Warsaw, Poland). Cells were grown under standard conditions (37°C, 5% CO_2_) in low glucose (1 mg/ml) Dulbecco's Modified Eagle Medium (DMEM) supplemented with 10% fetal bovine serum, 100 units/mL of penicillin, 100 μg/mL of streptomycin, and 25 μg/mL of amphotericin B (Antibiotic-Antimycotic).

### Western blotting

Adherent, alive cells were harvested into Laemmli SDS sample lysis buffer, sonicated and centrifuged at 10 000 × g for 10 min. Concentration of proteins was estimated by the BCA method; 100 mM DTT and 0.01% bromphenol was added to lysates before separation by SDS-PAGE (8, 12 and 15% gels were used). Total protein concentrations were determined using bicinchoninic acid (BCA) protein assay kit, according to manufacturer's instruction.The same protein amount (7.5 to 50 μg) was loaded into each well. Membranes were blocked in 5% non-fat milk dissolved in TBS containing 0.1% Tween-20 for 1 hour at room temperature (RT). Then, membranes were probed overnight at 4°C with antibodies specific for: p53, H2A.X and p21Cip1 (1:500 in TBS/ 5% milk), PARP-1, p62 and γ-H2AX (1:1 000 in TBS/ 5% milk), p-p53 (Ser15) (1:500 in TBS/ 5% BSA), p-S6 (1:1 000 in TBS/ 5% BSA), LC3B (1:5 000 in TBS/ 5% BSA). GAPDH (1:30 000 in TBS/ 5% BSA) was used as a loading control. Then proteins were detected using appropriate secondary HRP-conjugated antibodies (1:10 000 in TBS/5% milk) and ECL reagents as recommended by manufacturer.

### Detection of senescence-associated β-galactosidase (SA-β-Gal)

SA-β-Gal activity was detected according to modified Dimri et al. [68]. Briefly, adherent, alive cells were trypsinized, fixed with 2% formaldehyde, 0.2% glutaraldehyde in PBS, washed, cytospined and exposed overnight at 37°C to a solution containing 1 mg/ml 5-bromo-4-chloro-3-indolyl-b-D-galactopyranoside, 5 mM potassium ferrocyanide, 150 mM NaCl, 2 mM MgCl_2_, and 0.1 M phosphate buffer, pH 6.0.

### Enzyme-linked immunosorbent assay (ELISA) for vascular endothelial growth factor (VEGF) and interleukin-8 (IL-8)

Concentrations of secreted proteins VEGF and IL-8 in the culture media and in murine serum were measured using a colorimetric ELISA according to the vendor's instructions. Tests were specific for human proteins. Results were normalized to total cell number.

### Clonogenicity assay and cell viability determination

After the 3rd doxo cycle, adherent, alive cells were re-plated into 6-well plates at a density of 1000 cells per well and cultured for additional 21 days. Medium was changed every seven days. Afterwards, the colonies were fixed and stained in a mixture of methanol and 0,5% crystal violet (1:1) for 60 minutes. Cells were rinsed with water, dried at RT, and clones were counted. Images of the colonies (corresponding to single wells of the multi-well dishes) were normalized by division to their counterparts containing only background (estimated as the local maximum of the polynomial fit). Colonies were segmented from the corrected images using the images with Otsu thresholding (two classes, variance criterion). Clustered (touching) colonies were separated using Euclidean distance transform, followed by watershed. The colonies were counted and their equivalent radii estimated from the respective areas. All image processing operations were executed with CellProfiler 2.1. Cell viability were assessed using Lactate dehydrogenase (LDH) release test according to the vendor's instructions. The results were normalized to the total cell number.

### Matrigel assay

To test whether senescent cells can form tumorospheres, after the 3rd doxo cycle, alive, adherent HCT116 cells were replated into 96-well plates fulfilled with 50 μl of Matrigel Matrix (Growth Factor Reduced). Matrigel was polymerized at least for 30 minutes in 37°C before seeding. Cells were seeded at a density of 1 000 cells per well and cultured for additional 21 days. Medium was changed every seven days. Images of the spheres were taken weekly using inverted Olympus DP73 microscope, working in transmitted light mode. Images of the spheres were corrected for background (polynomial fit). The corrected images were processed with uniform filter (11 × 11 pixels) and subjected to gray-scale opening (radius 17 pixels). The sphere candidates were segmented with Otsu thresholding and the objects with circularity below 0.45 rejected from further analysis. The areas corresponding to the segmented spheres were quantified and expressed as radii of equivalent discs. All image processing operations were implemented using CellProfiler 2.1.

### DNA content analysis

For DNA analysis alive, adherent cells were collected by trypsinization, fixed in 70% ethanol and stained with propidium iodide (PI) solution (3.8 mM sodium citrate, 50 mg/ml RNAse A, 500 mg/ml PI, in PBS). Histograms of cellular DNA content were collected with Becton-Dickinson FACS Calibur flow cytometer and CellQuest software Pro 6.0 (log scale). The histograms were aligned using maximum corresponding to 2C population as the reference. At least 10 000 events were analyzed for each sample. The 1D gates of the 2C, 4C, 8C and 16C populations were set manually at the FWHM of the respective peaks of the and used consistently throughout the analysis.

### Side population (SP) evaluation

Alive, adherent cells were trypsinized, washed twice in PBS and then re-suspended at 10^6^ cells/ml in PBS, then incubated at 37°C with 5 μg/ml Hoechst 33342 (H33342). For the inhibitor control, 50 μM Verapamil was added 5 minutes prior to H33342. Cells were incubated for 90 minutes at 37°C with constant mixing. Staining was halted by rinsing in ice-cold PBS. Prior to measurement cells were stained with PI (4 μM) for viability assessment. Cells were analyzed on the BD LSRFortessa flow cytometer. Cells were excited with a UV laser (355 nm, 20 mW). The emission was detected through 450/50 nm BP (“Hoechst Blue”) and 530/30 BP with dichroic 505 LP (“Hoechst Red”) filters. All data were collected in linear mode and analyzed using BD FACSDiva 6.2 software. Cells were displayed on dot plots gated on live cells (PI negative) and viewed in a Hoechst Blue versus Hoechst Red dot plot to visualize the SP. The SP gate was determined by the use of the 50 μM Verapamil inhibitor-treated cells. Data for 30 000 cells was collected and analyzed.

### Flow cytometry analysis of cancer stem cell markers

Cells labeled with H33342 for SP were probed with specific fluorochrome-conjugated antibodies. Briefly, 1 × 10^5^ cells were incubated with 100 μl of PBS containing 0.1 μg of antibodies: APC-conjugated anti-CD133, FITC-conjugated anti-CD24 and AlexaFluor 700-conjugated anti-CD44 antibodies for 30 minutes on ice. Labeled cells were re-suspended in PBS and stained with PI (4 μM) for viability assessment. Isotypic IgGs served as a negative control. Flow cytometry was performed using a BD LSRFortessa instrument. Signal excited with 488 nm laser (50 mW): FITC and PI were detected in 530/30 and 575/26 BPs respectively; with 640 nm laser (40 mW): APC and AlexaFluor 700 in 670/14 and 730/45 BPs. Data for 30 000 cells was collected and analyzed using BD FACSDiva 6.2 software.

### Aldehyde dehydrogenase (ALDH) activity

Cells labeled with H33342 were resuspended at 10^6^ cells per milliliter in Aldefluor assay buffer with BODIPY-aminoacetaldehyde (BAAA) at a concentration of 1.5 μM for 30 min at 37°C. For the inhibitor control, 50 μmol/l of the specific ALDH inhibitor diethylaminobenzaldehyde (DEAB) was added. After washing with cells Aldefluor assay buffer were stained with 7-AAD (4 μM) for viability assessment. Flow cytometry was performed using a BD LSRFortessa instrument equipped with a 488-nm argon laser. Aldefluor fluorescence was detected using the green fluorescence channel (530/15 nm). 7-AAD fluorescence was detected using the orange fluorescence channel (585/21 nm). Data for 30 000 cells was collected and analyzed using BD FACSDiva 6.2 software.

### Fluorescent staining of lysosomes

Living cells were incubated with 1 μM LysoTracker^®^ Red DND-99 directly added to the culture medium for 30 minutes at 37°C. Nuclei were stained with the vital H33342 at 1 μg/ml for 5 minutes at 37°C and analysed using fluorescent Nikon Eclipse 50i microscope, CCD Evolutions VF camera (MediaCybernetics) and the Image-Pro Plus 6.0 software. Afterwards, cells were trypsinized, washed with PBS and immediately measured using a BD FACS Calibur and the CellQuestPro 6.0 software. Results are presented as percentage of control mean fluorescence in channel FL2; 10 000 events were analyzed for each sample.

### Fluorescent staining with Dil to determine slow-cycling cells

Living cells were incubated with Dil directly added in the culture medium at 37°C at 1 μg/ ml for 30 minutes. For flow cytometry analysis cells were stained on the day 1st and at the given time points were trypsinized, washed with PBS and immediately measured using a BD FACS Calibur and the CellQuestPro 6.0 software. Results are presented as percent of control mean fluorescence in channel FL2. 10 000 events were analyzed for each sample. For spheroid-forming analysis cells were stained on the day 14th and immediately seed into matrigel.

### Immunofluorescent staining

Cells grown on chamber slides were fixed with 4% formaldehyde for 10 minutes, washed in PBS, permeabilized with 0.5% Triton X-100 in PBS for 20 minutes and blocked for 60 minutes in PBS with 5% goat serum and 0.1% Triton X-100. Afterwards, cells were incubated with the primary antibodies at 1:100 (1:500 for LC3B) in the blocking solution at 4°C overnight in humidified chamber. After that samples were washed with PBS and incubated with secondary antibodies conjugated with AlexaFluor 488 or AlexaFluor 555 diluted 1:200 in PBS for two hours in RT in humidified chamber. Then the slides were stained for 5 minutes with H33342 (1 μg/ml in PBS) and mounted with Fluorescent Mounting Medium. In the control experiments, the steps with primary antibodies were omitted. Slides were analyzed using a fluorescent Leica DM4000B. Images were captured using Image Pro-Plus 5.0 software.

### Detection of Ki67^+^ cells and quantification of lysosomal activity with image cytometry

Series of optical sections were registered using DSD2 spinning disc confocal system (Leica/Andor) equipped with a 20× oil immersion objective (NA = 1.4), sCMOS camera (Andor Zyla 5.5) and 200W metal halide light source (Andor AMH-200-FS6). Fluorescence of H33342 (nuclei) was excited in 370–410 nm range and detected in 430–475 nm range. Fluorescence of AlexaFluor 488 (Ki67) was excited in 469–497 nm range and detected in 500-550 nm range. Fluorescence of LysoTracker^®^ Red DND-99 was excited in 545–560 nm range and detected in 565–600 nm range. The fluorescence signal digitized with 12 bit precision and the image acquisition parameters (time, gain) were set so as to fill 65 % the dynamic range. Single optical section comprised 2124 × 1171 pixels (corresponding to 0.1 × 0.1 μm each) and the spacing between the sections was 0.7 μm. The 3D images (H33342, AlexaFluor 488, LysoTracker^®^ Red DND-99) were converted to 2D, with average z-projection, and used in further processing steps, implemented with CellProfiler (http://cellprofiler.org/). The H33342 images were subjected to average filtering (9 × 9 kernel) and used to calculate the background (local minima within 75 × 75 pixel blocks). Following background subtraction the candidate nuclei were segmented with Otsu thresholding (two classes, weighted variance criterion). The identified regions were split with Euclidean distance transform followed by watersheding (the minimum object distance of 45 pixels). The regions which were characterized by circularity < 0.35 or area < 1000 pixels were excluded from further analysis. 2D images of Ki67 were corrected with background subtraction (local minima within 90 × 90 pixel blocks) and processed with tophat filter (25 × 25 pixels). Regions of high Ki67 fluorescence intensity were identified with background thresholding, followed by watersheding (the minimum object distance of 7 pixels). These Ki67-positive regions were assigned to corresponding nuclear masks. The nuclei containing one or more such regions were regarded as Ki67-positive, otherwise as negative. 2D images of LysoTracker^®^ Red DND-99 were corrected with background subtraction (local minima within 90 × 90 pixel blocks) and processed with tophat filter (7 × 7 pixels). Regions of high LysoTracker^®^ Red DND-99 fluorescence intensity (lysosomes and their clusters) were identified with constrained Otsu thresholding followed by watersheding. The groups of lysosome masks were assigned to single cells with Voronoi tessellation. The fluorescence intensity of lysosomes was then quantified on the cell-by-cell basis from the background-corrected LysoTracker^®^ Red DND-99 images. The cells were assigned to classes according to the number (none, low, high) and average fluorescence intensity (0, low, high) of the corresponding lysosomes.

### Long-term time-lapse

Cells were seeded in 6-well plate and starting on the day 17th of AFTER CHEMO protocol they were recorded for 60 hours. Pictures were taken every 10 minutes using time-lapse Leica AF7000 microscopy in DIC Nomarski contrast with 10 × objective, using microscope equipped with DFC350 FX camera (Leica, Mannheim, Germany), and environmental chamber (Pecon, Erbach, Germany).

### Inoculation of tumor cells and *in vivo* assessment of tumor growth

To determine an ability of senescent cells to form tumors, senescent HCT116 (*N* = 7) and BAF A1-treated senescent HCT116 cells (*N* = 7) were injected subcutaneously into 6-week old female NOD.CB17-Prkdc *s c i d* /NCrHsd mice (Harlan Laboratories S.r.l., Italy). Untreated HCT116 (*N* = 6) were used as a control. Alive, adherent cells were harvested on the day 14th. Each subcutaneous injection consisted of 5 × 10^3^ cells together with 50 μL of Matrigel Matrix mixed with 50 μL of PBS. The mice were kept in a specific pathogen-free environment and checked for tumor development during 2 months. Once a week the animals were weighed and tumor sizes were documented. Tumor volumes were determined by means of caliper and calculated using the following formula: *V* = *D* × *d*^2^ × 0.5 (*V* is the tumor volume, *D* is the biggest dimension; *d* is the smallest dimension). The mice were then euthanized by isoflurane inhalation followed by cervical dislocation. The tumors were excised, fixed in 10% formalin overnight and subjected to routine histological hematoxylin and eosin (H&E) staining. The study was approved by the Local Ethics Committee for Animal Experimentation at the Nencki Institute of Experimental Biology.

### Measurement of HUVEC proliferation

Proliferation of HUVECs was measured in cells cultured in 96-well plates using BrdU incorporation assay according to vendor's protocol. To measure angiogenic properties of HCT116, 1.5 × 10^3^ HUVECs were seeded in 100 μl of complete DMEM medium. Medium was replaced with incomplete DMEM medium (without FBS) 24 hrs later. After 24 hrs of starvation HUVEC cells were treated with the mixture of 50 μl of incomplete DMEM medium and 50 μl of conditioned medium collected from BAF A1-treated senescent HCT116 cells on the day 17th. Media were preincubated with VEGF-neutralizing antibodies or istotype control antibodies for 30 minutes, at 37°C, with mixing. BrdU incorporation assay was performed after 48 hours.

### Statistical analysis

*In vitro* experiments were performed in duplicates or triplicates and repeated at least three times. Results are shown as means ± SE. *P*-value was calculated by student's type-2 two-tailed *t* test.

## SUPPLEMENTARY FIGURES AND MOVIE




