# Supplementary material for: Bafilomycin A1 triggers proliferative potential of senescent cancer cells in vitro and in NOD/SCID mice
Source: Oncotarget. 2016 Dec 21;8(6):9303–22. doi: 10.18632/oncotarget.14066 (PMC5354733; doi:10.18632/oncotarget.14066)
Supplement: Supplementary file 1 [file oncotarget-08-9303-s001.pdf]

Bafilomycin A1 triggers proliferative potential of senescent cancer cells *in vitro* and in NOD/SCID mice

Supplementary Materials

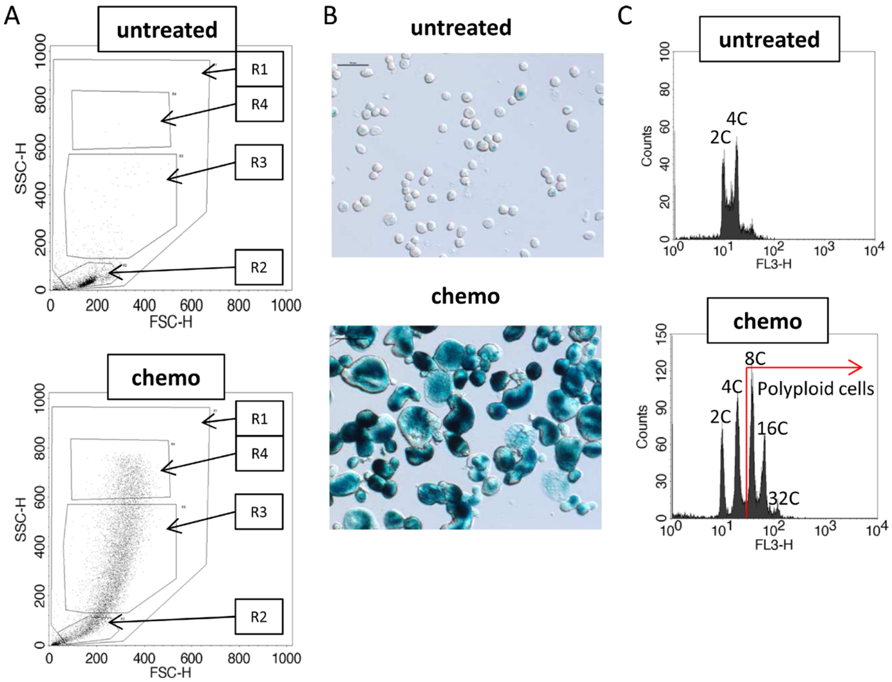

Supplementary Figure S1: HCT116 cells treated with CHEMO protocol show features of senescence. (**A**) Visualization of representative dot-plots showing gating strategy of granular cells used in experiments presented in Figures. 1C, 3E and Supplementary Figure S4B. Cells treated with the CHEMO protocol were displayed in FSC/SSC mode using flow cytometry and gated as follows: gate R1 - all cells (cell debris were excluded), gate R2 - non-granular cells (based on untreated cells), gate R3-medium-granular cells, gate R4 - high-granular cells. (**B**) Representative photos of SA-β-gal staining on untreated and CHEMO-treated cells. Staining was performed on cytospined cells to enable easier quantification of SA-β-gal positive cells. Original magnification 200×. Data obtained by light microscopy. Scale bar–100 µm. (**C**) Visualization of representative histograms showing cell cycle analysis. Cells were stained with PI and DNA content was determined by flow cytometry. Data was collected in logarithmic mode. Similar way on analysis was used in Figure 1E and for ploidy discrimination with vital H33342 presented in Figures 6 and 7B.

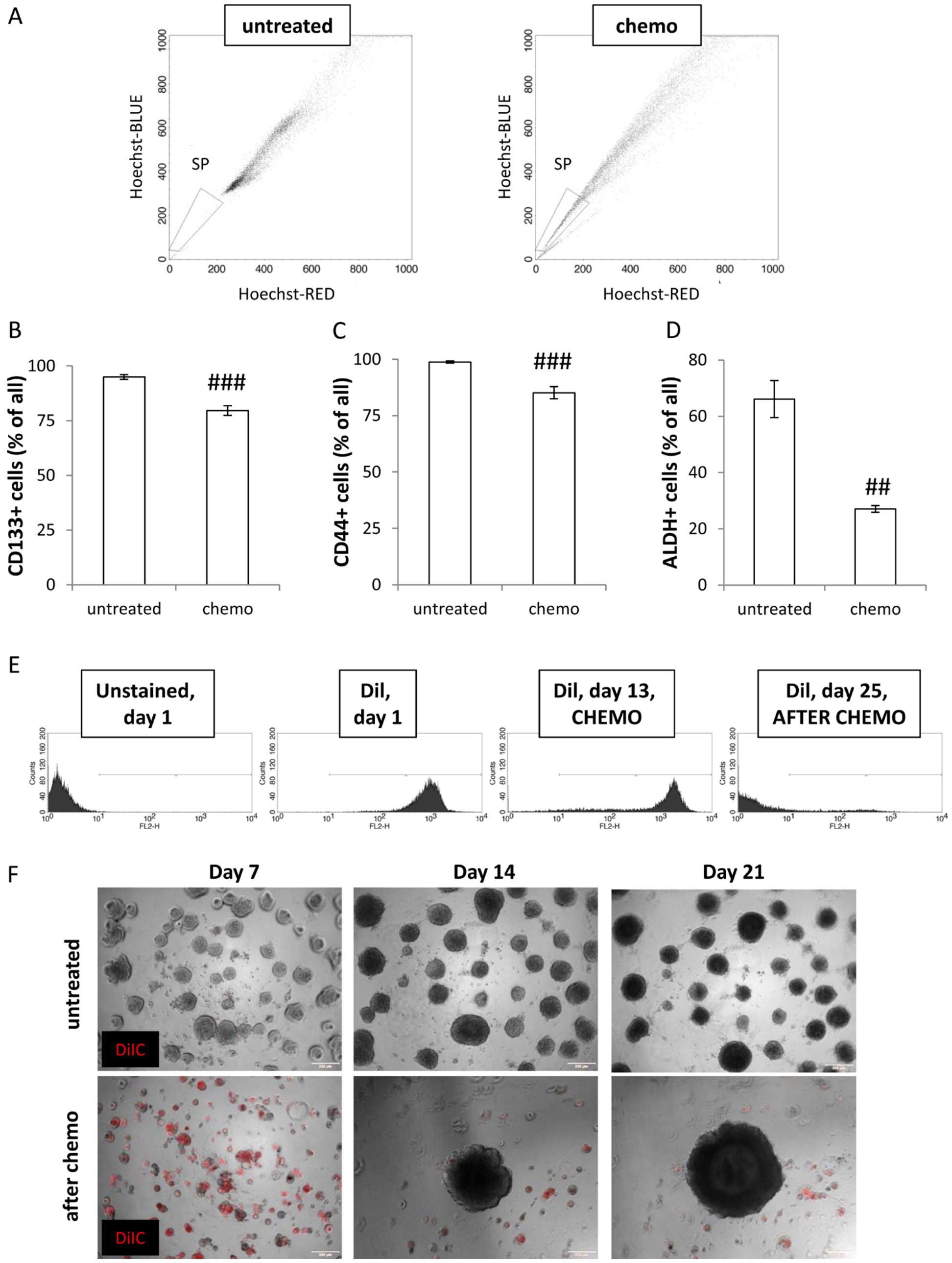

Supplementary Figure S2: Senescent HCT116 cells exhibit certain properties of cancer stem cells. (**A**) Visualization of representative dot-plots showing evaluation of side population (SP) by flow cytometry. Untreated and doxo-treated cells were labeled with H33342 and excited with a UV laser (355 nm, 20 mW). The emission was detected through 450/50 nm BP ("Hoechst Blue") and 530/30 BP with dichroic 505 LP ("Hoechst Red") filters. Data was collected in linear mode. (**B**–**C**) Evaluation of percentages of CD133 (B) and CD44 (C) positive cells. Cells were probed with anti-CD133-APC (B) and anti-CD44-AlexaFluor700 (C) antibodies and percentages of positive cells were determined by flow cytometry. Cells labeled with isotypic IgGs were used as a negative control. Cells were treated with the CHEMO protocol. (**D**) Evaluation of percentages of HCT116 cells that display ALDH activity. Cells co-incubated with DEAB, an ALDH inhibitor, served as a negative control. Cells were treated with the CHEMO protocol. (**E**) HCT116 cells were stained with fluorescent dye Dil on the 1st day. Percentages of cells retaining Dil was determined with flow cytometry, at every time point. Unstained cells were used as a negative control. The representative histograms are shown. (**F**) Untreated and cells treated with AFTER CHEMO protocol were stained with Dil on the 14th day and seeded into matrigel – 1 000 cells into 96-well plate. Medium was replaced every week. Representative photos of spheroids taken 7, 14 and 21 days after seeding are presented. Dil positive cells are visualized as red. Original magnification 100×. Data obtained by light and fluorescent microscopy. Scale bar-100 µm. $^{\#}p < 0.05$, $^{\#\#}p < 0.01$, $^{\#\#\#}p < 0.001$ - untreated vs. CHEMO. Each bar represents mean ± SEM, $N \geq 3$.

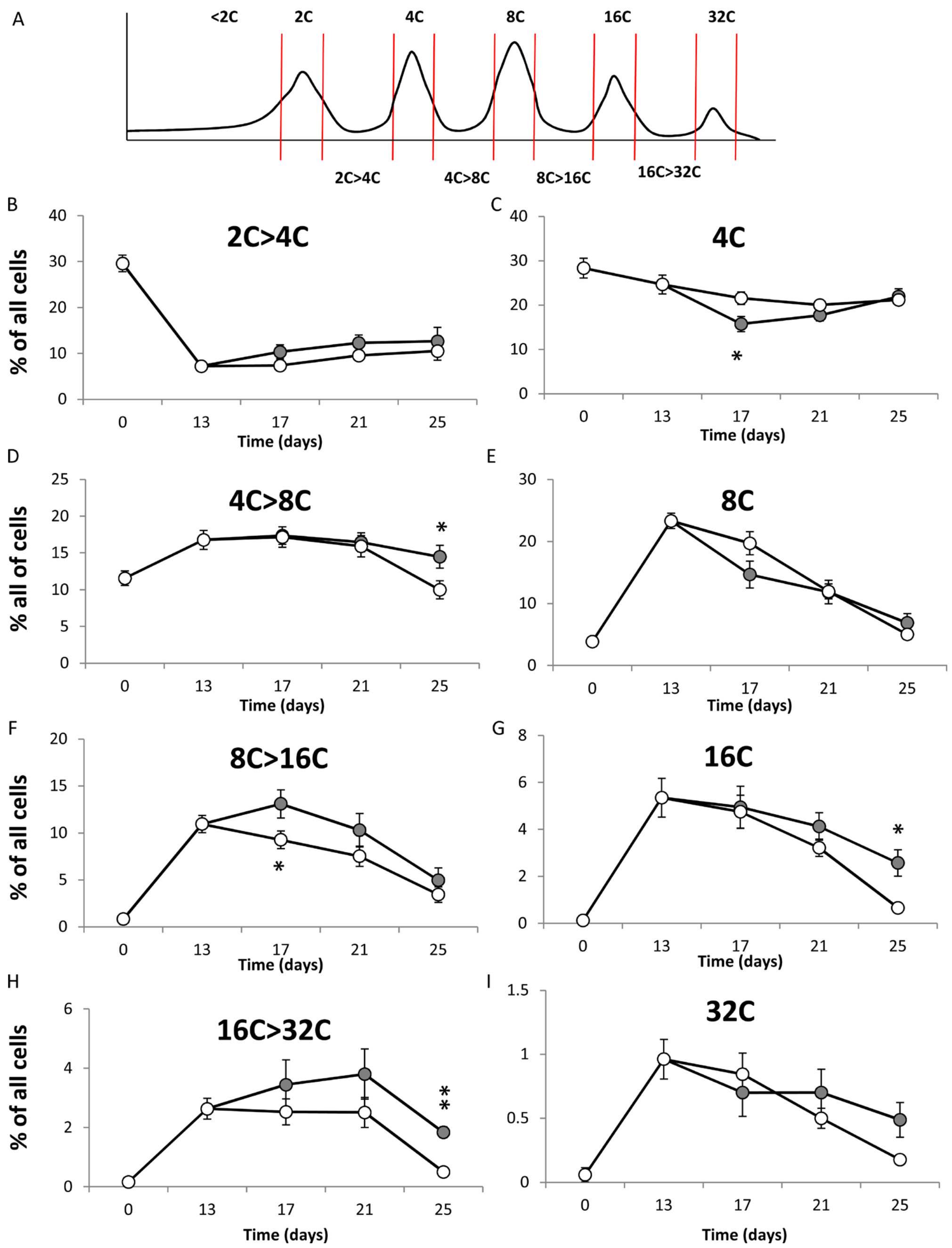

Supplementary Figure S3: A single pulse of BAF A1 increases percentages of doxo-treated HCT116 in polyploid state. Cells were treated with the AFTER CHEMO or the AFTER CHEMO + BAF A1 protocol. (**A**) Detailed analysis of cell cycle. Analysis was performed using PI staining and flow cytometry. Evaluation of percentage of cells with: 2C > 4C (**B**), 4C (**C**), 4C>8C (**D**), 8C (**E**), 8C > 16C (**F**), 16C (**G**), 16C > 32C (**H**), 32C (**I**) DNA content. Each bar represents mean ± SEM, $N \geq 3$.*$p < 0.05$, **$p < 0.01$, ***$p<0.001$ – AFTER CHEMO vs. AFTER CHEMO + BAF A1.

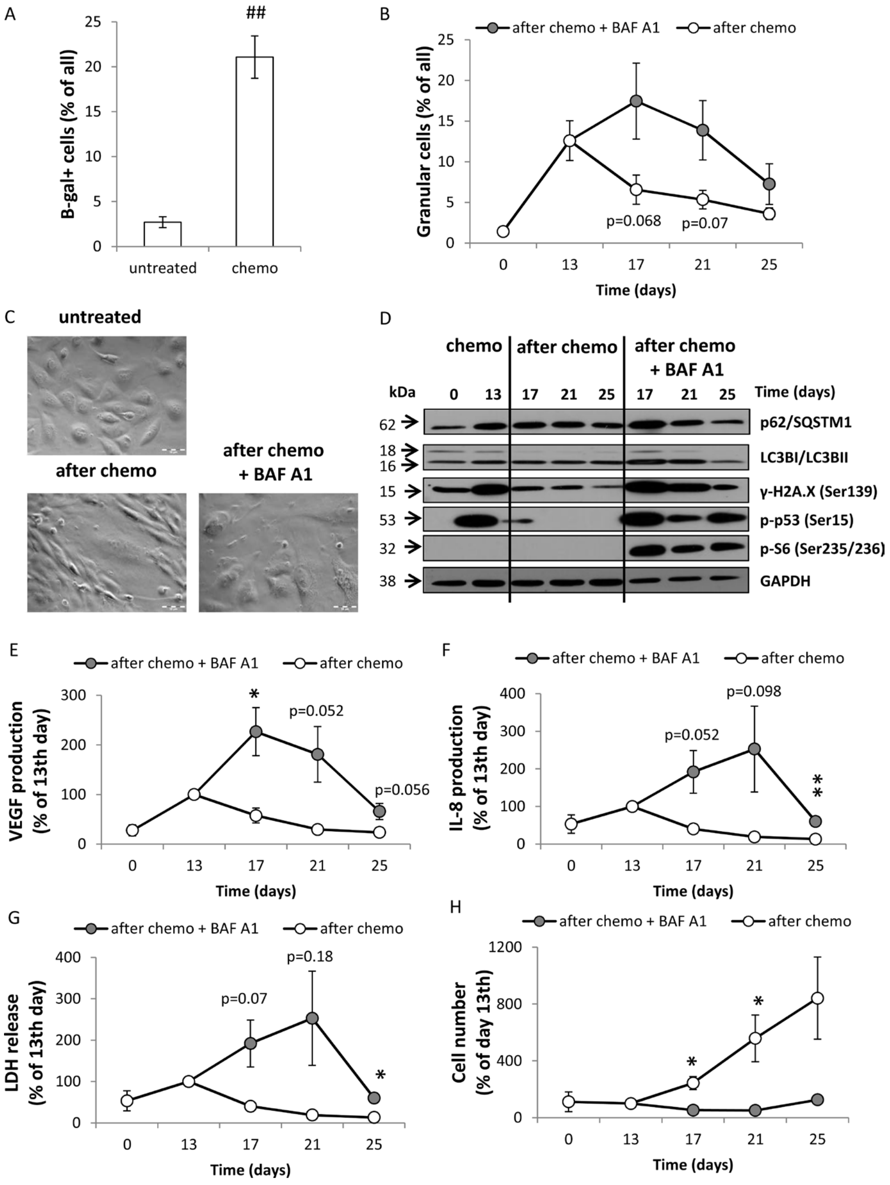

Supplementary Figure S4: A single pulse BAF A1 treatment maintains doxo-treated glioblastoma LN18 cells in a senescent state and delays culture re-population. (**A**) Quantification of SA-β-gal^{+} cells in untreated and doxo-treated cultures on the 13th day (CHEMO). Detection of senescent cells using SA-β-gal staining on cytospined cells. Data obtained by light microscopy. (**B**) Quantification of percentages of granular cells using FSC/SSC analysis by flow cytometry. (**C**) Representative photos show morphological alterations in CHEMO-treated LN18 cells. Original magnification 200×. Data acquired by light microscopy. Scale bar - 50 μm. (**D**) Representative blots show expression of autophagy-related proteins: LC3B II/I and p62, as well as DDR and geroconversion proteins: γ-H2A.X, p-p53, and p-S6. Detection of GAPDH served as a loading control. (**E**–**F**). Secretion of SASP cytokines: VEGF (E) and IL-8 (F) in supernatants harvested from treated cultures was determined by colorimetric ELISA. Results were normalized to total cell number counted in Bürker's chamber. (**G**) Evaluation of cell mortality was performed using LDH assay. Results were normalized to total cell number counted in Bürker's chamber. (**H**) Evaluation of cell number in various times. Each bar represents mean ± SEM, $N = 4$.*$p < 0.05$, **$p < 0.01$, ***$p < 0.001$ – AFTER CHEMO vs. AFTER CHEMO + BAF A1.

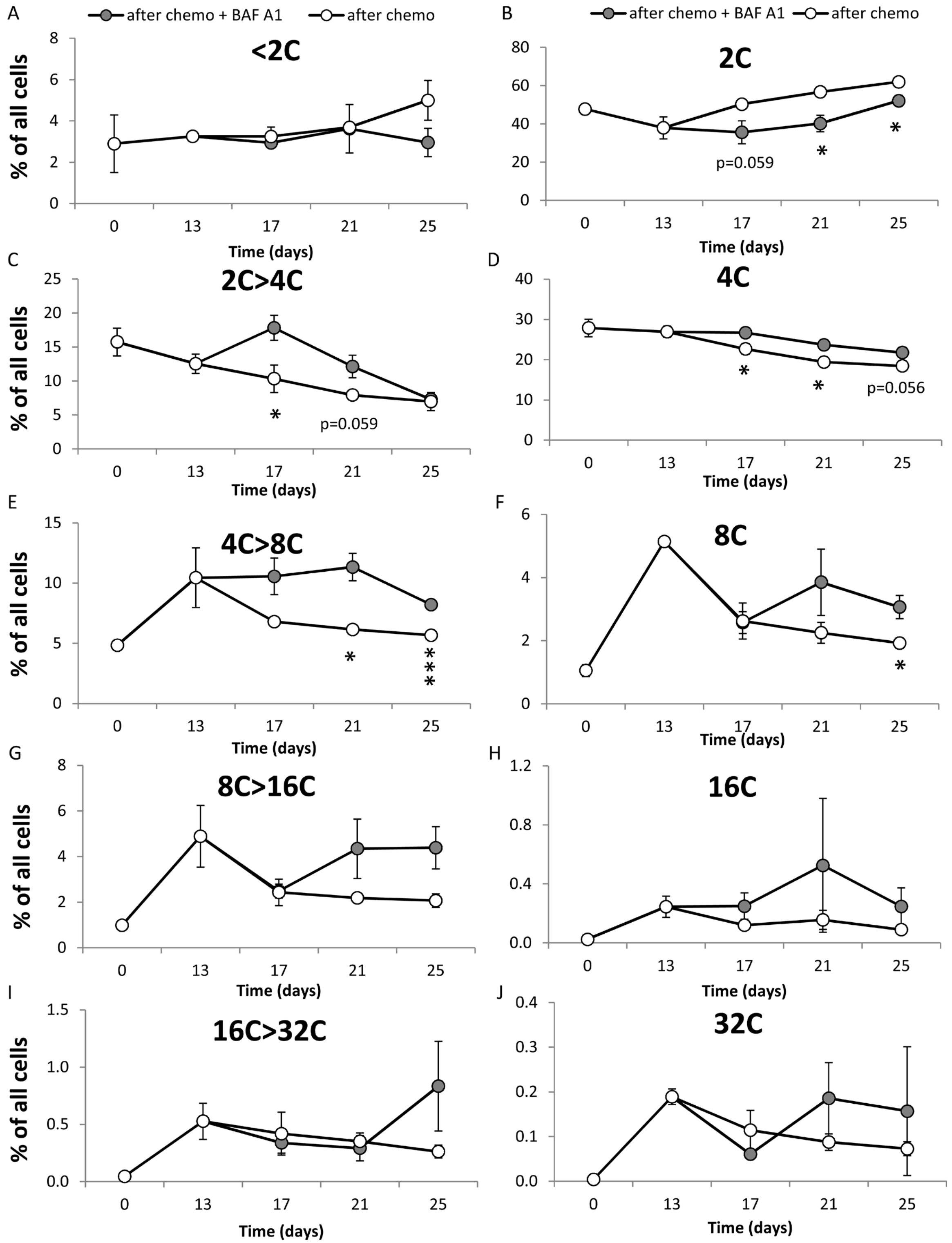

Supplementary Figure S5: A single pulse of BAF A1 increases numbers of polyploid cells in doxo-treated LN18 cells. Detailed analysis of cell cycle in cultures exposed to the AFTER CHEMO or the AFTER CHEMO + BAF A1 protocol was performed using PI staining and flow cytometry (similar way of analysis was used in Supplementary Figure S3). Evaluation of percentages of cells with: < 2C (**A**), 2C (**B**), 2C > 4C (**C**), 4C (**D**), 4C > 8C (**E**), 8C (**F**), 8C > 16C (**G**), 16C (**H**), 16C > 32C (**I**), 32C (J.) DNA content. Each bar represents mean ± SEM, $N = 4$.*$p < 0.05$, **$p < 0.01$, ***$p < 0.001$ – AFTER CHEMO vs. AFTER CHEMO + BAF A1.

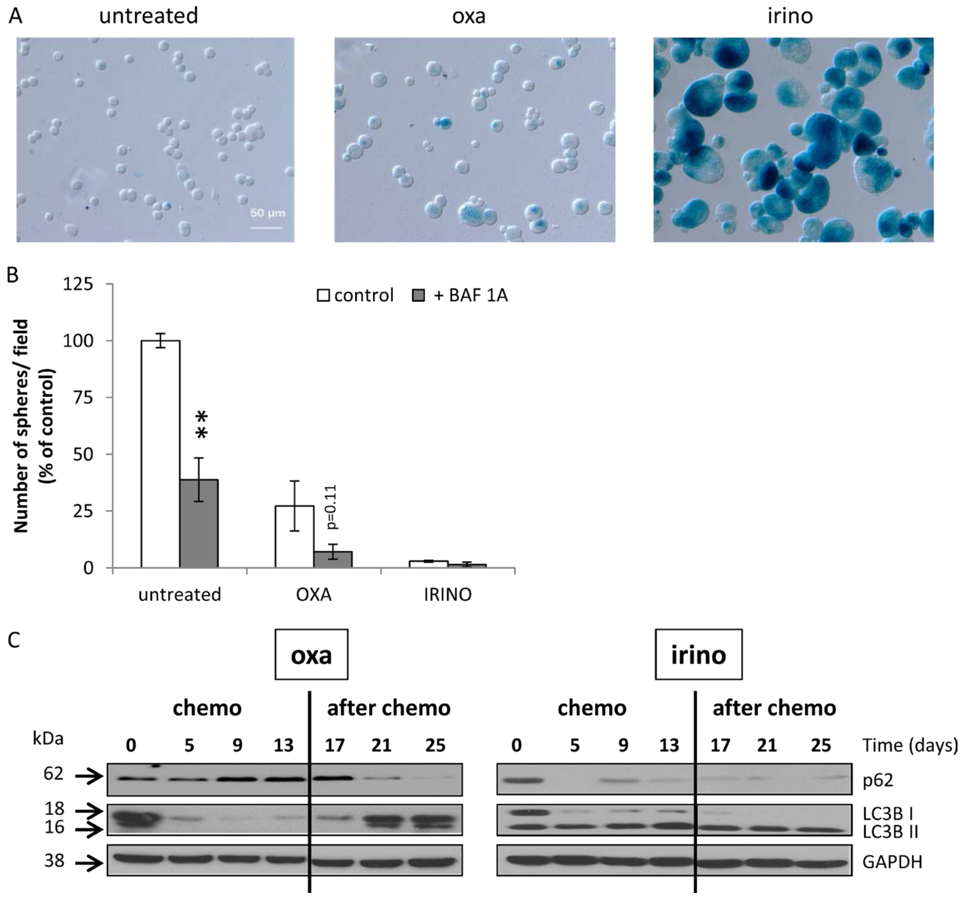

Supplementary Figure S6: A single pulse of BAF A1 does not affect irino-treated HCT116 cells, but affects oxa-treated cells. HCT116 ells were subjected to 2.5 µM irinotecan (irino) or 5 µM oxaliplatin (oxa) according to the CHEMO protocol. (**A**) Representative photos of SA-β-gal staining on untreated, oxa- or irino-treated cells on the 13th day (CHEMO). Staining was performed on cytospined cells. Original magnification 200×. Data obtained by light microscopy. Scale bar - 100 µm. (**B**) Evaluation of numbers of colonies formed by controls or cells treated with the AFTER CHEMO or the AFTER CHEMO + BAF A1 protocol. Drug-treated cells (1 000 cells/6-well plate) were seeded on the 14th day just after BAF A1 removal. Medium was replaced every week. Representative photos were taken three weeks after seeding. (**C**) Representative blots show expression of autophagy-related proteins: LC3B II/I and p62. GAPDH served as a loading control. Each bar represents mean ± SEM. $N \geq 3$. *$p < 0.05$, **$p < 0.01$, ***$p < 0.001$ – AFTER CHEMO vs. AFTER CHEMO + BAF A1.

Supplementary Movie S1: Senescent HCT116 cells produce viable, proliferating progeny. Cells were seeded in 6-well plate and treated with the AFTER CHEMO protocol. Cells had being recorded starting from the 17th day. Pictures were taken every 10 minutes for the next 60 hours using time-lapse microscopy in DIC Nomarski contrast with 10× objective, equipped with environmental chamber. See Supplementary_Movie_S1
